# Study on the dry shrinkage characteristics and size effect of swell-shrink characteristic soil

**DOI:** 10.1371/journal.pone.0307679

**Published:** 2024-08-05

**Authors:** Aijun Chen, Xu Zhang, Xiong Shi, Shanshan Zhao, Junhua Chen

**Affiliations:** School of Architecture and Traffic Engineering, Guilin University of Electronic Technology, Guilin, Guangxi, China; Al Mansour University College-Baghdad-Iraq, IRAQ

## Abstract

Swell-shrink characteristic soils exhibit a high susceptibility to cracking during the drying process, which poses a significant risk of various geological disasters. Among these, the occurrence of drying shrinkage acts as a prerequisite for the cracking phenomenon. Therefore, it is of utmost importance to comprehend the specific characteristics associated with the drying shrinkage mechanism. To investigate the drying shrinkage behavior of swell-shrink characteristic soils, a series of drying shrinkage experiments were conducted on long strip samples of red clay and expansive soil. Utilizing three-dimensional digital image correlation (DIC) technology, the surface displacement, strain, and anisotropic shrinkage rates of the soil samples during the drying process were obtained, and the size effect on the drying shrinkage of swell-shrink characteristic soil were analyzed. The research findings are as follows: The displacement development of the soil samples in the X and Y directions can be divided into two stages: a linear growth stage and a stable displacement stage. In the Z direction, the soil surface deformation can be divided into three stages: soil surface arching, vertical shrinkage, and shrinkage stabilization. The drying shrinkage of swell-shrink characteristic soil exhibits anisotropy, with the vertical shrinkage rate being the largest, followed by the longitudinal and then the transverse directions. Additionally, the soil sample shrinkage exhibits a size effect, whereby the shrinkage rates in all directions increase with increasing sample width and thickness. During the drying shrinkage process, the stress state on the soil surface evolves from initial tensile strain to subsequent compressive strain. The strain at different positions and times within the soil sample is not uniform, resulting in the non-uniformity and anisotropy of the sample shrinkage. This study provides important insights into the cracking mechanism of swell-shrink characteristic soils and serves as a valuable reference for related laboratory experiments, which will contribute to better prediction and control the geological hazards caused by the drying shrinkage of swell-shrink characteristic soils.

## Introduction

Soil serves as the principal medium for a variety of geological engineering activities undertaken by humans and is a widely used construction material. Throughout history, humans have extensively utilized soil in various engineering. Currently, soil finds widespread application in diverse fields, including civil engineering, mining, and agriculture, and plays a pivotal role in these endeavors. Prolonged exposure of soil to dry climatic conditions often leads to uneven volumetric shrinkage deformation and the formation of surface cracks. This phenomenon is particularly noticeable in swell-shrink characteristic soil, which exhibit significant swelling upon absorbing water and subsequent shrinkage upon losing moisture [1]. Examples of swell-shrink characteristic soils include high-plasticity clays such as red clay and expansive soil. Such soil types are highly sensitive to changes in their internal moisture content and substantial moisture loss can result in considerable shrinkage deformation [2]. Regions with prevalent swell-shrink characteristic soils often experience various geological engineering disasters due to soil shrinkage. For instance, soil shrinkage can alter air-filled porosity (AFP) of the soil matrix, thereby impacting plant growth and agricultural productivity [3]. Cracks resulting from soil dry shrinkage can compromise soil structural integrity, facilitate rainwater infiltration, accelerate soil slope instability, and trigger geotechnical disasters such as landslides and mudslides [4–6]. In the context of nuclear waste processing, a rapid temperature rise in the surrounding area can cause the backfill soil, used for buffering and containment, to shrink rapidly, This, in turn, can lead to the immersion of groundwater containing radioactive elements, causing damage to the local ecological environment and human health [7]. Additionally, the covering layer of urban garbage sanitary landfills may crack due to evaporation-induced shrinkage, thereby diminishing its barrier function and increasing the risk of contaminant leakage [8]. A flood control dam in Hungary experienced significant shrinkage deformation from 1989 to 1993, substantially increasing the risk of hazardous situations and threatening the safety of local residents [9]. Soil shrinkage and cracking have implications in various geotechnical engineering fields, including soil mechanics, foundation engineering, geotechnical hazard mitigation, and environmental geotechnology. Investigating the fundamental mechanism of cracking in cohesive soils can help develop strategies to mitigate the occurrence of such geological engineering disasters, with soil drying shrinkage being a primary causal factor. Therefore, studying the drying shrinkage behavior of cohesive soils holds both theoretical and practical significance in preventing and controlling geotechnical engineering disasters.

In recent years, the rapid industrial development and global temperature rise have led to the frequent occurrence of extreme high-temperature events, which has brought the issue of soil drying-induced shrinkage to the forefront of scholarly attention [10, 11]. In the study of soil drying shrinkage, laboratory experiments are commonly employed, but there is no unified standardized for the specimen’s the shape and size, which has introduced certain difficulties in the comparison and practical application of research findings. Existing studies have revealed that soil samples exhibit anisotropic shrinkage behavior. For instance, when the specimen is in the form of a long strip, the vertical shrinkage is greater than the horizontal shrinkage, and the higher the initial water content, the more pronounced the shrinkage [12, 13]. When the soil sample is cylindrical, the axial shrinkage rate is larger than the radial shrinkage [14, 15]. It has been observed that as the initial thickness of the soil sample increases, the shrinkage time gradually lengthens, and the shrinkage rate gradually increases [16, 17]. Additionally, when the specimen size differs, the shrinkage rate also varies, indicating a significant size effect on the soil shrinkage behavior. The size effect refers to the phenomenon where the material properties change with the alteration of the material dimensions [18]. In the context of this study, the size effect specifically refers to the relationship between the shrinkage characteristics of swell-shrink characteristic soils and the sample size during the drying shrinkage process. However, systematic research on the size effect of expansive soil samples is currently lacking, and it is necessary to further investigate the shrinkage characteristics of soil samples of different sizes during the drying process to provide more reliable references for engineering practice.

Soil displacement is a critical measurement parameter that quantifies the deformation of soil samples during the drying shrinkage process. Deformation within expansive soil is typically non-uniform, meaning that different positions may undergo varying displacements and strains, which is an important consideration. Analyzing the displacement of different parts allows for the identification of the heterogeneity of deformation within the soil. Additionally, studying the strain on the soil surface helps understand the deformation occurring during the drying shrinkage process, including soil volume reduction and surface crack formation. Besides crack formation on the surface, the drying shrinkage process of expansive soil can also induce internal cracks. Studying the displacement of different parts enables tracing the formation and expansion process of cracks and understanding their distribution, direction, and depth. This comprehension is crucial for evaluating soil stability and the integrity of engineering structures built on soils. Thus, analyzing soil sample displacement and strain during the shrinkage process provides valuable insights into the mechanisms underlying shrinkage anisotropy and size effects. However, current research primarily focuses on measuring the overall soil shrinkage in different directions, with few studies investigating displacement changes at various points on the soil surface throughout the entire shrinkage process. Moreover, research on strain changes of soil samples in different parts throughout the entire shrinkage process is lacking.

Uneven soil deformation during drying presents challenges for volume measurement. Direct measurement methods like steel rulers or vernier calipers can easily damage samples, causing significant measurement errors. Volume displacement method also disrupt the sample’s drying state and hinder accurate recording of volume change. However, advancements in imaging technology are lowering measurement costs, leading to more scholars adopting image-based techniques for sample measurement. For example, in the study of shrinkage and expansion deformation of cohesive soils, particle image velocimetry (PIV) technology offers continuous and precise measurement of soil displacement [19]. Digital Image Correlation (DIC) has also garnered attention from scholars due to its high accuracy, non-contact nature, speed, and dynamic capabilities, being applied in various domains. In the field of crack detection, DIC has demonstrated its effectiveness in detecting cracks on civil structures such as buildings and slabs [20, 21]. Moreover, in the characterization of high-performance materials, DIC technology has been used to study mechanical behaviors like damage and fracture of composite materials in complex coupled environments in-situ [22]. The key advantage of DIC technology is its ability to analyze and compare relevant information from two sets of two-dimensional digital images to derive the three-dimensional displacement field of the sample [23, 24]. This makes DIC a well-established technical approach for monitoring specimen displacement throughout the drying shrinkage process.

The study conducted dry shrinkage experiments on Hunan red clay and Guangxi expansive soil, coupled with Digital Image Correlation (DIC) technology, to analyze the displacement, strain, and anisotropic shrinkage characteristics of these two types of swell-shrink characteristic soils. The aim was to deeply investigate the shrinkage behavior and size effect of swell-shrink characteristic soils, which hold important reference value for research on the development mechanism of soil cracking. Currently, laboratory studies on soil drying shrinkage lack a unified standard for sample size and shape. This research systematically carried out drying shrinkage experiments on soil samples of different sizes, providing a reference for improving the related experimental methods and enhancing the comparability and universality of the research results.

## Materials and methods

### Characterization of test soil

**Fig 1:**
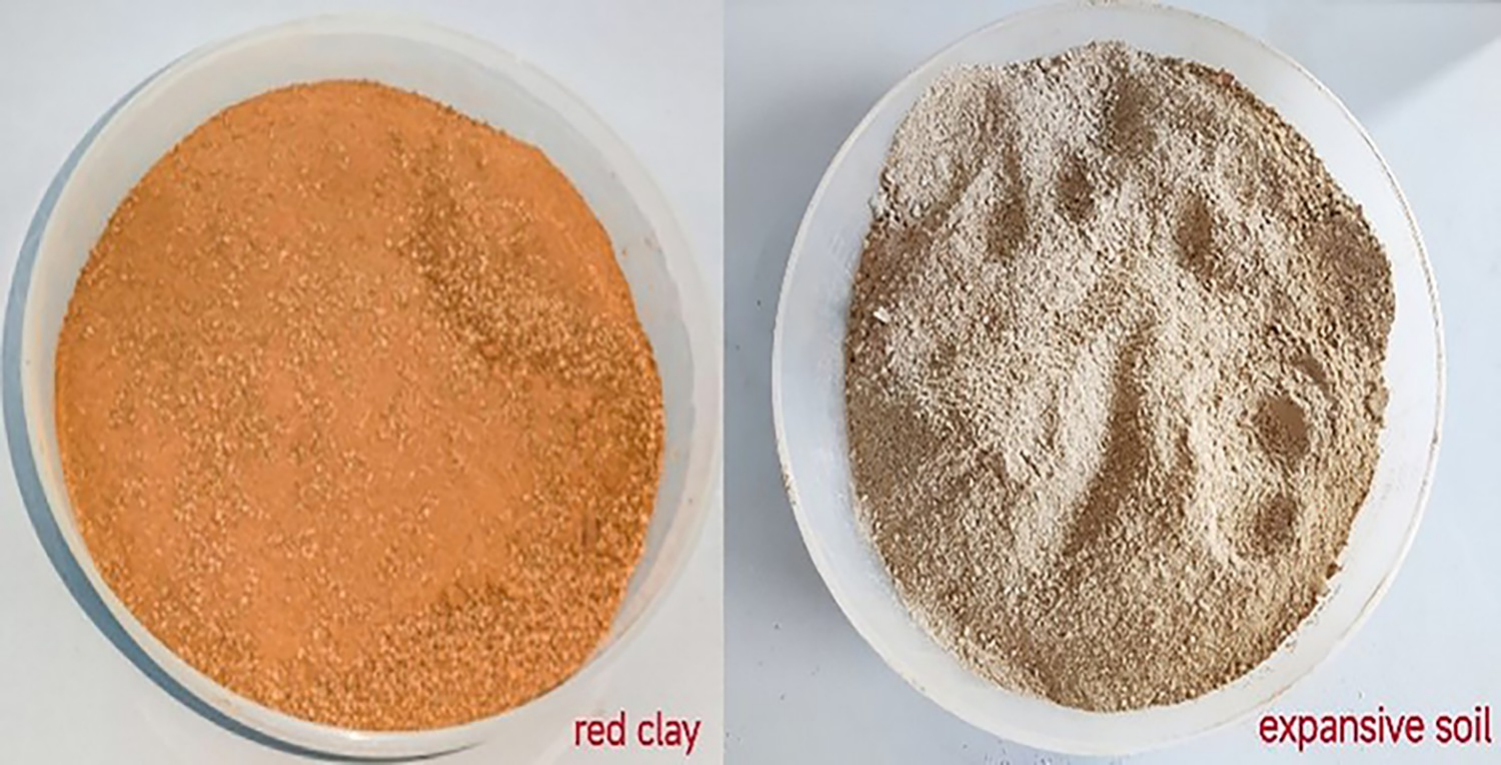
Red clay (left) and expansive soil (right).

The experiment utilized Hunan red clay collected from the Shaoyang area. The soil was excavated from a depth of 2.5 to 4.0 meters below the surface and displays a brown color. Table 1 summarizes the basic physical properties of the Hunan red clay. The study utilized expansive soil sourced from the Nanning area of Guangxi. The soil was excavated from a depth of 1.5 to 4.5 meters below the surface and exhibits a light yellow appearance. Table 2 presents the basic physical properties of the Guangxi expansive soil. Fig 1 displays photographs of the red clay (left) and expansive soil (right) samples for reference.

**Table 1:** The basic physical property indexes of red clay.

Physical Properties	Value
Specific gravity (g/cm^3^)	2.72
Liquid limit (%)	67.7
Plastic limit (%)	28.3
shrinkage limit (%)	17.8
Plasticity index	39.4
Optimum moisture content (%)	18.5
Maximum dry density (g/cm^3^)	1.86
Clay (%)	62.8

**Table 2:** The basic physical property indexes of expansive soil.

Physical Properties	Value
Specific gravity (g/cm^3^)	2.71
Liquid limit (%)	47.6
Plastic limit	20.2
shrinkage limit (%)	28.5
free expansion rate (%)	51.7
Plasticity index	27.4
Optimum moisture content (%)	17.2
Maximum dry density (g/cm^3^)	1.78

The particle composition and plasticity index analysis from Tables 1 and 2 reveals that the Hunan red clay belongs to the category of high liquid limit clay. Furthermore, the Guangxi expansive soil exhibits a free expansion rate of 51.7%, which classifies it as weakly expansive according to the ’Technical Specification for Building in Expansive Soil Areas’ [25], a national standard in China. It is noteworthy that both the Hunan red clay and Guangxi expansive soil demonstrate swell-shrink characteristics.

### Preparation of soil samples

The soil samples were first air-dried. The air-dried soil was then crushed using a rubber mallet and passed through a 2mm sieve. The air-dried soil was subsequently mixed with water to prepare soft soil samples with targeted moisture contents. Specifically, the red clay samples were prepared at 50% moisture content, while the expansive soil samples were prepared at 35% moisture content. These moisture contents were selected to be equal to 75% of the respective liquid limits of the soil types. After thoroughly mixing the soil and water, the soft soil samples were sealed and cured for 24 hours to ensure a uniform moisture distribution throughout the samples. Following the curing process, the transparent sample molds were retrieved, and their inner surfaces were lined with plastic film. This measure was taken to reduce the friction between the soil and the mold walls. The soft soil samples were then carefully placed into the prepared molds, compacted by vibration to achieve a consistent density, and the soil surface was evenly covered with a thin layer of lime powder. The lime powder was applied to mask the original soil color, facilitating the subsequent digital image analysis. Additionally, a random distribution of coal powder was sprinkled on top of the lime powder to create speckled patterns, which would aid in tracking the movement and deformation of soil particles during the experiments. The rationale for using the lime and coal powders is that the fine particles of the expansive soil and red clay render the soil texture visually indistinct. Without the application of these marker materials, it would be challenging to accurately capture the surface characteristics and particle-level behaviors using digital image processing techniques. The size of the lime and coal powders, typically in the range of a few micrometers, has been demonstrated to have a negligible impact on the overall soil behavior [26]. Ensuring a consistent moisture content within the soil samples was a critical aspect of the experimental design. Conducting repeated experiments with identical moisture contents and dimensions across different samples would be highly challenging, as minor variations could lead to inconsistent results. However, the deviation of individual samples is not expected to significantly influence the overall patterns and trends observed in the experiment, thus preserving the representative nature of the study.

For this experiment, five samples of red clay and five samples of expansive soil were prepared, each set having different dimensions. By varying the sample sizes, the aim was to investigate the size effect on the swelling and shrinkage behavior of these expansive soils. It is important to note that, when determining the sample dimensions, consideration was given to the limited observation range of the DIC technique. To ensure the feasibility of the subsequent DIC observations, the sample lengths were not made excessively long. Referring to the sample lengths used in the previous study by Tang et al. [27], the sample lengths in the current experiment were set to 20 cm. Additionally, to ensure the samples had a near- long strip shape, the ratio of sample height to length, as well as the ratio of sample width to length, were constrained to not exceed 4. However, if the sample height and width were less than 1 cm, it would have resulted in operational difficulties and low experimental precision. The red clay samples are denoted by "C," and the expansive soil samples are denoted by "E." The specific sample dimensions are presented in Table 3.

**Table 3:** Test grouping.

numbering	Long(mm)	Wide(mm)	High(mm)
C1/ E1	200	10	10
C2/ E2	200	30	10
C3/ E3	200	50	10
C4/ E4	200	30	30
C5/ E5	200	30	50

### Experimental procedure

**Fig 2:**
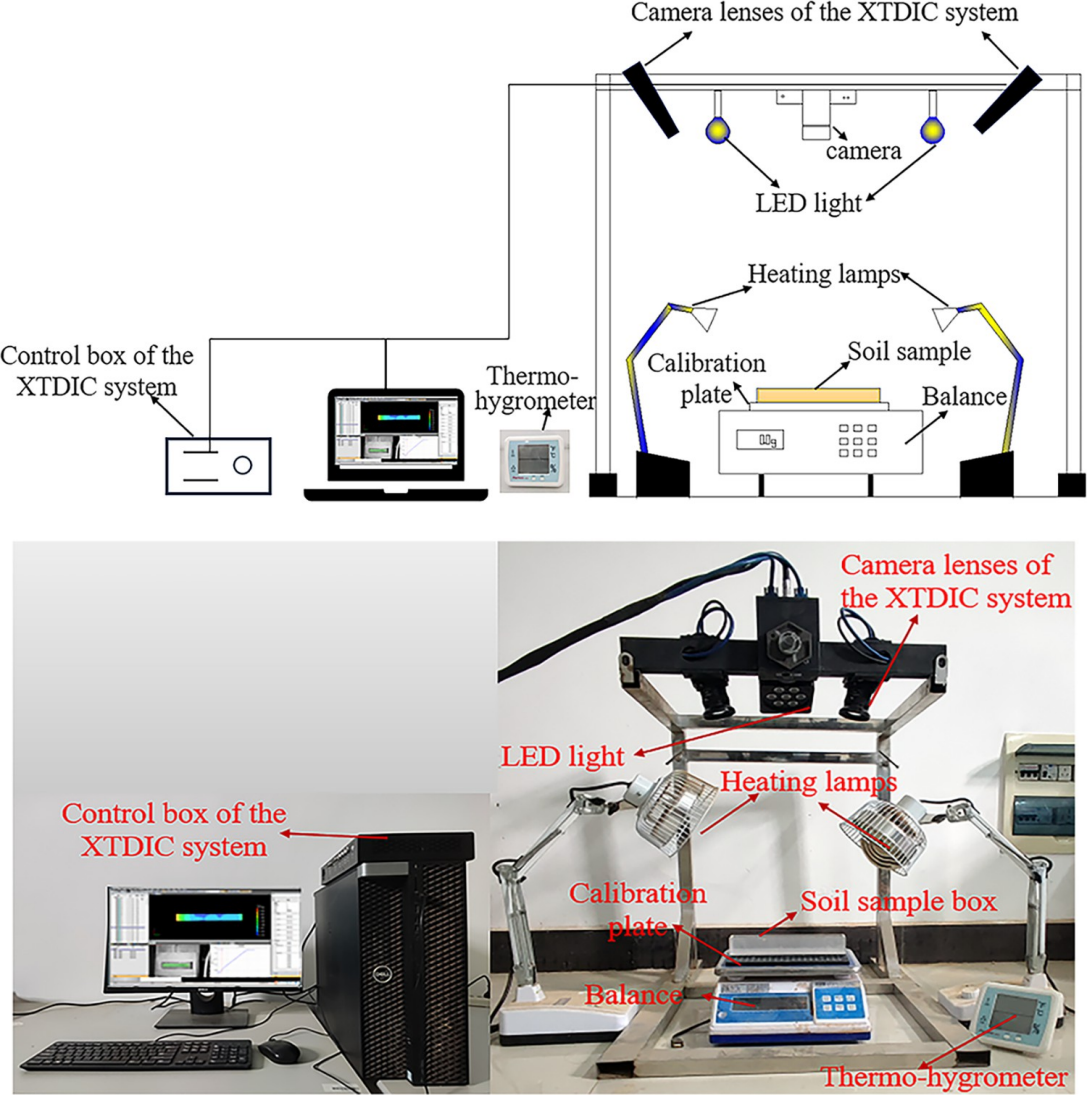
Schematic diagram of the test device.

This study employed a custom drying apparatus, with the primary equipment being the 3D Deformation Measurement System (XTDIC) developed by Xi’an Xintuo 3D Optical Measurement Technology Co, Ltd. XTDIC is an optical, non-contact three-dimensional deformation measurement system that integrates digital image correlation (DIC) technology and binocular stereo vision technology. It is capable of capturing the three-dimensional coordinates, displacement, and strain of the soil surface during the drying process. The apparatus comprises an electronic balance, two 230W solar lights, two high-precision camera lenses, an LED light source, and a DIC device. Fig 2 depicts the schematic diagram of the apparatus.

Before the experiment commences, the soil sample is positioned on an electronic balance. Two light sources are directed towards the soil sample to induce water evaporation and soil shrinkage. The laboratory maintains a consistent ambient temperature of (22 ± 1)°C and relative humidity around 60% ± 2%. At the onset of the experiment, the XTDIC system and high-precision cameras capture the initial state of the soil sample simultaneously. The electronic balance, cameras, and DIC system are synchronized with a recording interval of 10 minutes. The experiment is concluded when the electronic balance indicates a weight change of less than 0.5g within an hour.

## Basic principles of the XTDIC system

**Fig 3:**
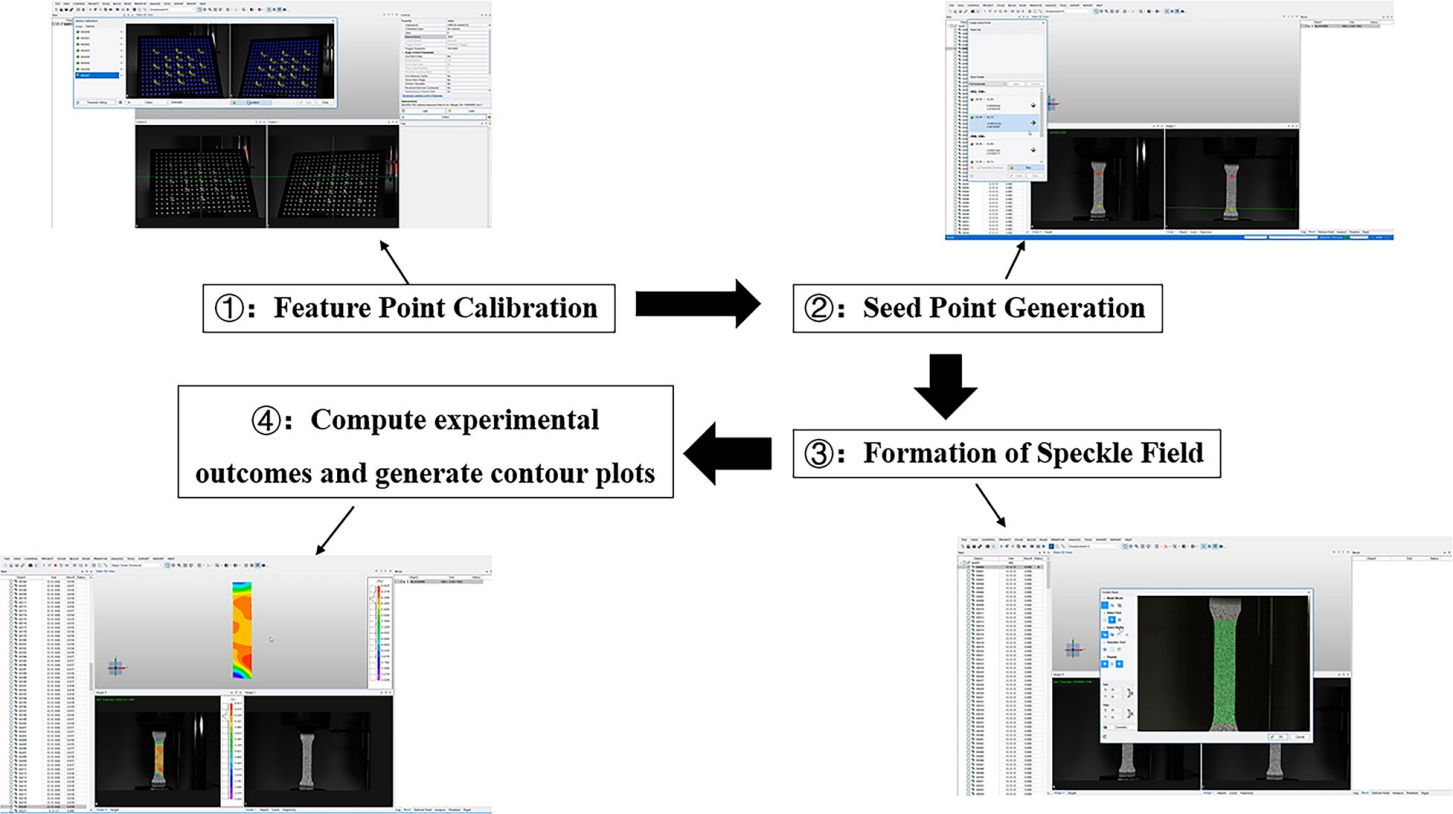
Flowchart of the operational steps of the XTDIC system.

**Fig 4:**
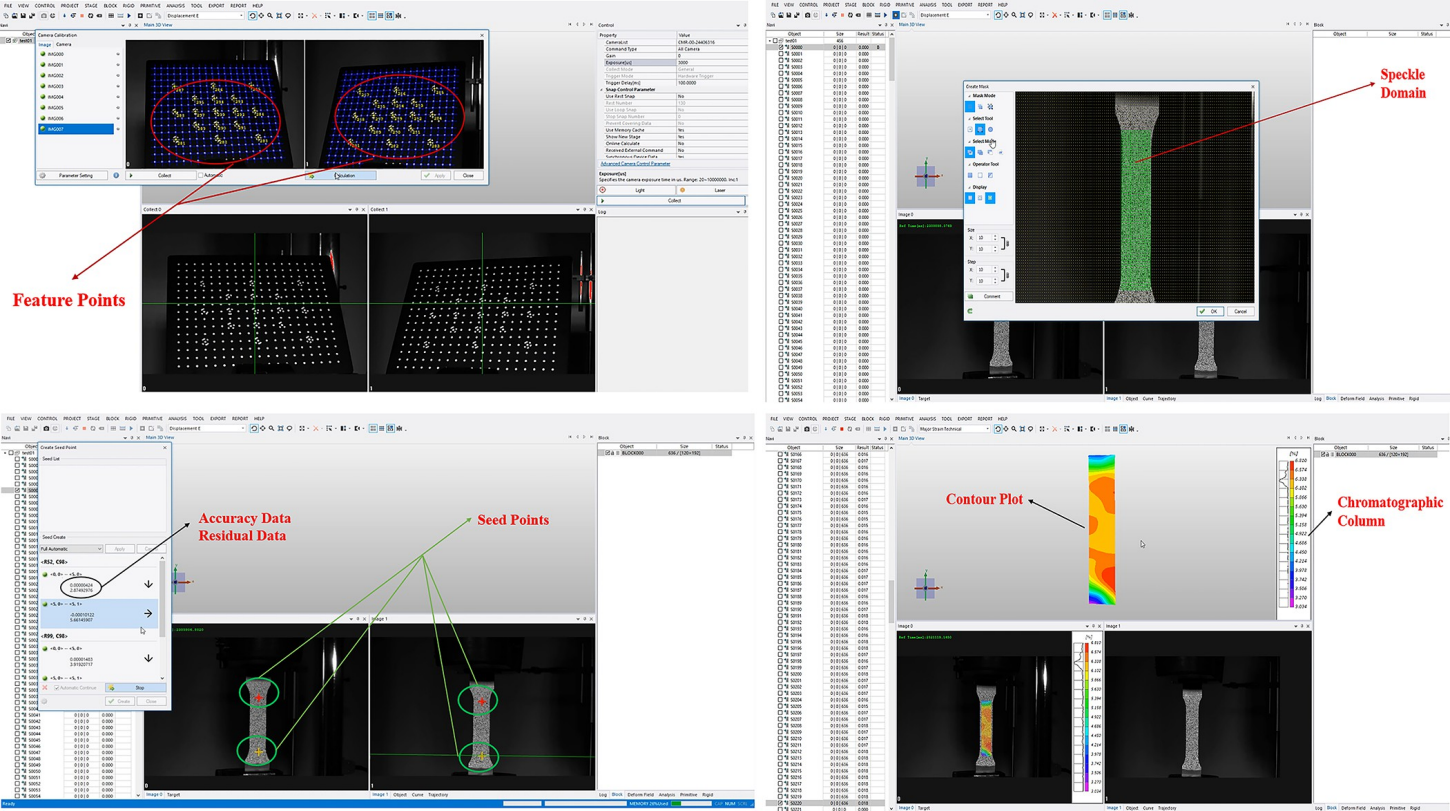
Detailed diagram of the operational steps of the XTDIC system. a. Feature Point Calibration. b. Seed Point Generation. c. Formation of Speckle Field. d. Compute experimental outcomes and generate contour plots.

The XTDIC system employed in this study integrates advanced stereoscopic vision technology and DIC analysis techniques. The system leverages DIC algorithms to precisely track and match the positions of discrete data points distributed across the surface of the soil specimen. The stereoscopic vision component then calculates the full three-dimensional coordinates of these tracked surface points based on their parallax values, which had been meticulously pre-calibrated prior to the experiment using established photogrammetric methods. By continuously recording the evolving 3D coordinates of the tracked points located within the designated deformation measurement region on the soil surface, the XTDIC system is able to derive a high-resolution displacement field. This displacement data can then be further processed using numerical differentiation techniques to compute the corresponding strain field manifested across the soil sample during the course of the experiment. The detailed step-by-step operational procedures for implementing the XTDIC system are comprehensively illustrated in Figs 3 and 4.

### Digital image-related technologies

The Digital image correlation (DIC) technique employed in this study operates by leveraging image analysis algorithms to track the displacement of discrete data points distributed across the surface of the soil specimen. This method subdivides the captured image into a grid of discrete pixel elements, with each pixel assigned a unique grayscale value corresponding to its brightness intensity. Consequently, the image data is transforms into a digital matrix that can be archived within the computer system.

Assuming that any point P(x_1_, y_1_) in the reference sub-image area corresponds to point Q(x_2_,y_2_) in the target sub-area after undergoing deformation, the positional relationship between point p and point q can be expressed as follows:

$$\begin{array}{l} x_{2}=x_{1}+\mu +\frac{\partial \mu }{\partial x}\Delta x+\frac{\partial \mu }{\partial y}\Delta y\\ x_{2}=y_{1}+v+\frac{\partial v}{\partial x}\Delta x+\frac{\partial v}{\partial y}\Delta y \end{array}$$ (1)

In this context, u and v represent the displacements of the center point of the reference image subregion along the X and Y axes respectively. The symbols $\frac{\partial_{\mu }}{\partial_{x}},\;\frac{\partial_{\mu }}{\partial_{y}},\;\frac{\partial_{v}}{\partial_{x}},\;\frac{\partial_{v}}{\partial_{y}}$ denote the first-order displacement gradients from the center point of the image subregion to any point (x, y) within the subregion. Additionally, *Δ_x_, Δ_y_* denote the distances from any point (x, y) in the reference image subregion to the center point of the same subregion.

In the course of this process, the acquisition of sample position information is accomplished by determining the first-order displacement gradient of u and v with respect to the image subregion. By tracking the movement of the test point, it becomes possible to determine the displacement and deformation across the entire image before deformation takes place.

### Binocular stereoscopic vision technology

**Fig 5:**
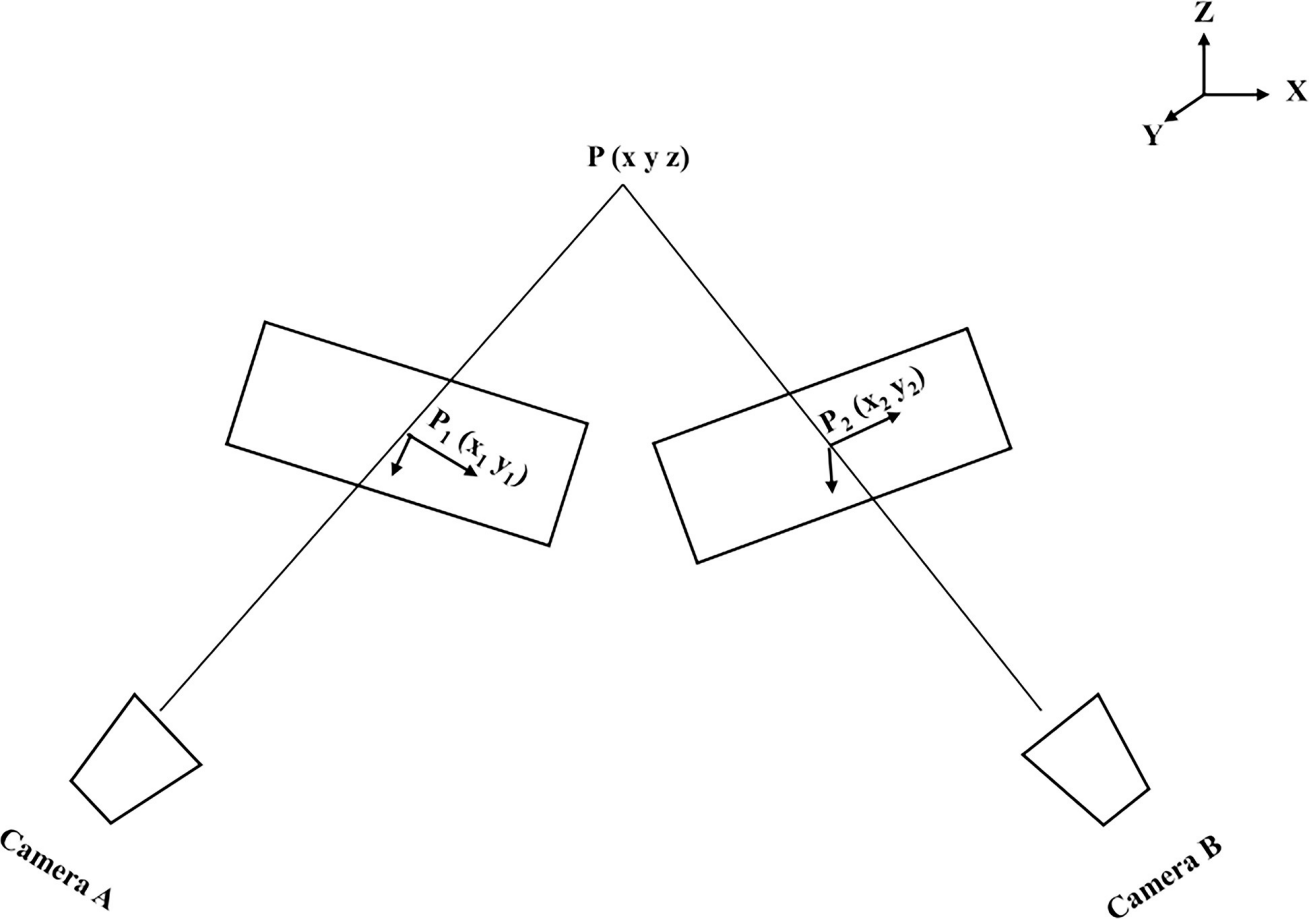
Binocular stereo vision schematic.

Conventional digital image techniques are limited to capturing two-dimensional displacement and strain information from multiple points on the surface of a soil sample. To obtain the three-dimensional coordinates of these points, a binocular stereo vision system consisting two cameras is required [28]. As illustrated in Fig 5, camera A captures the plane coordinate information P_1_ (x_1_, y_1_) of point P, while camera B captures the plane coordinate information P_2_ (x_2_, y_2_) of the same point P. In the three-dimensional coordinate system, let the coordinates of point P be P (x, y, z). Since points P, P_1_, and P_2_ lie on the same plane, we can derive the three-dimensional coordinate information of point P.

## Results and discussion

### Soil surface displacement

**Fig 6:**
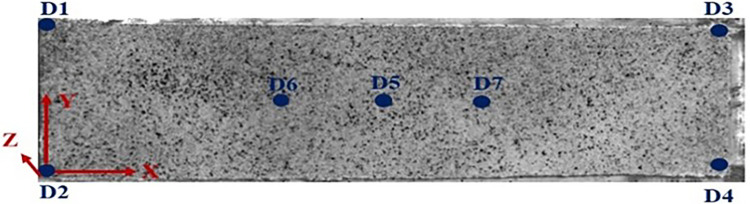
Schematic diagram of the orientation of each point.

This study investigates the displacement variations on the surface of two soil samples, namely C3 and E3, chosen as representative examples. Fig 6 illustrates the coordinate system of the soil sample surface and the locations of each point. The four corner points of the soil sample surface are labeled as D1-D4, while D5-D7 represent the center points of the soil sample along the Y-axis. It is worth noting that soil sample E3 follows the same labeling and positioning as soil sample C3.

**Fig 7:**
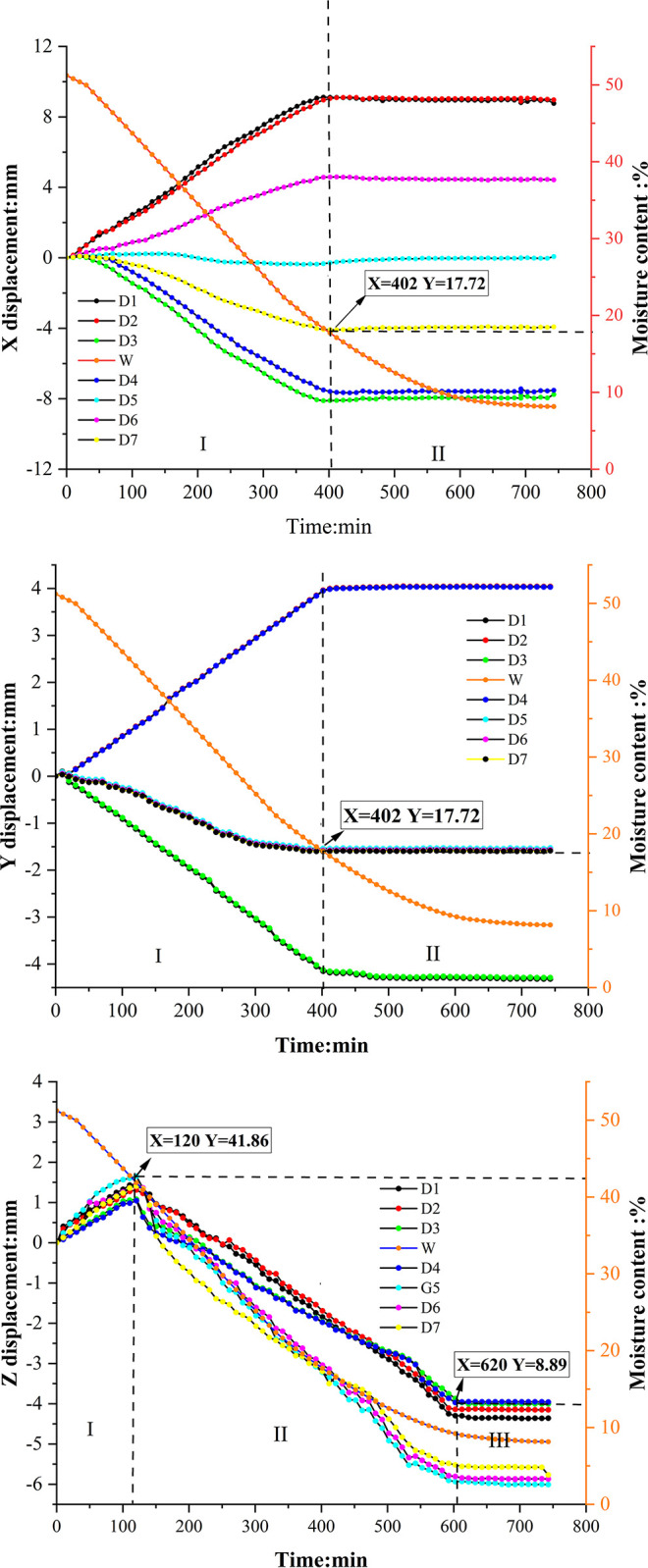
Displacement in all directions on the surface of red clay. a. (X direction). b. (Y direction). c. (Z direction).

**Fig 8:**
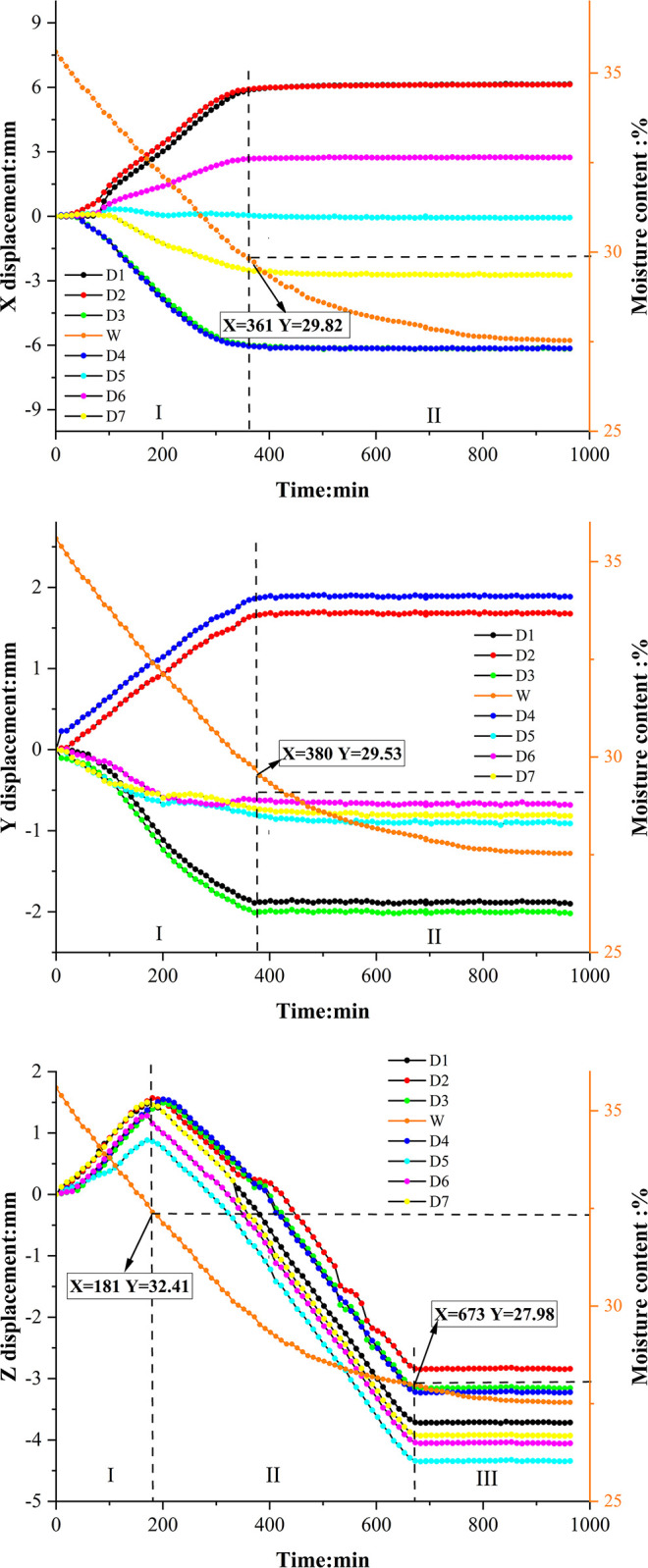
Displacement in each direction on the expansive soil surface. a. (X direction). b. (Y direction). c. (Z direction).

Soil sample C3 takes 750 minutes to dry, while soil sample E3 requires 960 minutes to drying. Figs 7 and 8 depict the time-dependent changes in the displacement of each point on the soil surface for both soil samples C3 and E3 in the X, Y, and Z directions. These figures also illustrate the variations in soil sample moisture content (W in the figures) throughout the entire testing process. Furthermore, the X-direction in this study is defined as the horizontal direction of the soil sample (D1→D3), representing its length. The Y-direction is defined as the vertical direction of the soil sample (D2→D1), indicating its width. As for the Z-direction, it is defined as the vertical direction of the soil sample, representing its height.

Analysis of Fig 7A and 7B reveals similar displacement patterns among various points on the surface of red clay soil sample C3 in both the X and Y directions, which can be divided into two stages. Initially, in the X direction, the soil sample undergoes linear growth from 0 to 402 minutes, during which surface points experience displacement, steadily increasing at rates between 0.01 mm/min and 0.02 mm/min. Subsequently, from 402 to 750 minutes, the displacement of surface points stabilizes, indicating the onset of a stabilization stage. During stabilization, the magnitudes of displacement in the X direction for D1, D2, and D6 (located on the left side of the soil sample) are 8.76 mm, 8.97 mm, and 4.42 mm, respectively, directed positively along the X-axis towards the center of the soil sample. Conversely, for D3, D4, and D7 (located on the right side of the soil sample), displacements in the X direction are 7.96 mm, 7.61 mm, and 3.95 mm, respectively, directed negatively along the X-axis towards the center of the soil sample. Notably, D5 experiences minimal displacement of only 0.02 mm. The displacement directions in the X direction vary between the positive and negative axes, with characteristic points on the left and right sides of D5 moving towards the center of the soil sample. D1-D4 represent the four corner points of the soil sample, with D1 differing from D2 by 0.21 mm and D3 differing from D4 by 0.35 mm due to their proximity along the X-axis. The displacement difference between D1 and D3 is 0.8 mm, and between D2 and D4 is 1.36 mm. It’s evident that when equidistant from the center in opposite directions, the displacement magnitudes are nearly equal during stabilization. D1 has a displacement 4.34 mm larger than D6, and D3 has a displacement 4.01 mm larger than D7, owing to their greater distance from the center. All points exhibit displacement towards the "center" of the soil sample, with closer points displaying smaller displacements. Comprehensive analysis suggests the presence of a contraction center in the X direction near point D5.

In the Y-direction, the 0 min—402 min period represents a linear growth stage, while the 402 min—750 min period exhibits a displacement stabilization stage. During the linear growth stage, the sample displays a growth coefficient ranging from 0.009 mm/min to 0.02 mm/min. When the Y-direction displacement reaches stability, the magnitudes of the displacements are as follows: D1 and D3 (located on the upper side of the soil surface) exhibit displacements of 4.31 mm and 4.28 mm, respectively, in the negative Y direction towards the center of the soil sample. D2 and D4 (located on the lower side of the soil surface) show displacements of 4.04 mm and 4.03 mm, respectively, in the positive Y direction towards the center of the soil sample. The displacements of D5-D7 are relatively minor, around 1.55 mm, in the negative Y direction, also towards the center of the soil sample. Among the four corner points D1-D4, the difference in displacement magnitude between D1 and D3 is 0.03 mm, while the difference between D2 and D4 it is only 0.01 mm. This small difference magnitude are due to the similar Y-coordinate values of these points. The comprehensive analysis of the Y direction displacements of D1-D7 indicates that the soil sample exhibits a contraction center in the Y direction, where the points closer to the center experience smaller displacements, and the displacement directions of all surface points point towards which is the contraction center of the soil sample in the Y direction.

The displacement patterns observed in expansive soil sample E3 in the X and Y directions are similar to those observed in red clay, although the durations of each phase differ. In the X direction, expansive soil E3 undergoes a linear growth phase from 0 to 361 minutes, followed by a stabilization phase from 361 to 960 minutes. Similarly, in the Y direction, the linear growth phase of expansive soil E3 extends from 0 to 380 minutes, with a subsequent stabilization phase from 380 to 960 minutes. It is worth noting that expansive soil achieves displacement stabilization earlier than red clay in both the X and Y directions. However, the complete stabilization of expansive soil requires a longer duration compared to red clay.

**Fig 9:**
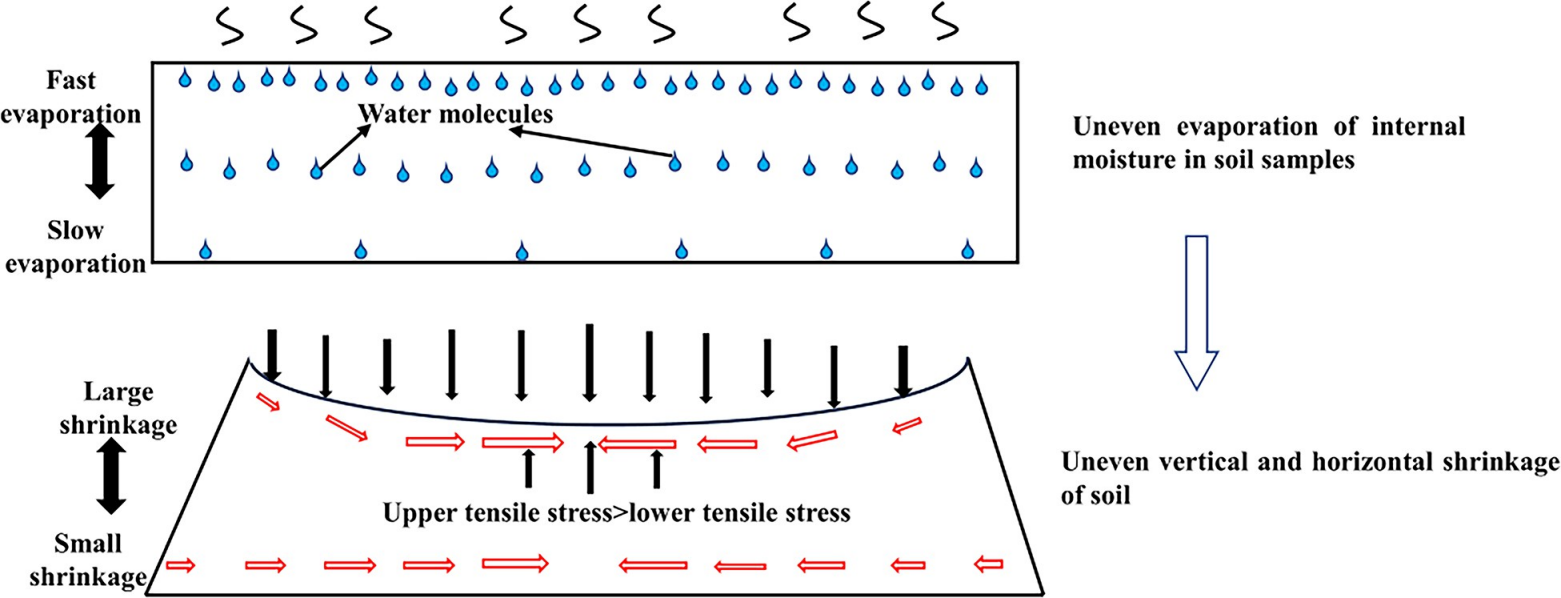
Schematic diagram of the stress on the soil sample during the drying process.

The displacement trends in the Z direction for both soil types, as depicted in Figs 7C and 8C, exhibit similarity. Examining red clay soil sample C3, the evolution of Z directional displacement evolution can be categorized into three phases: in the initial stage of the drying test, spanning from 0 to 120 minutes, all points on the soil surface undergo displacement towards the positive Z direction, resulting in a slight arching of the soil surface. At this stage, the soil sample experiences a rapid increase in temperature, but significant evaporation of water has not yet occurred. Subsequently, between 120 and 620 minutes, displacement occurs in the negative Z direction, indicating vertical settling of the soil. Finally, from 620 to 750 minutes, all points stabilize, signifying completion of the shrinkage process. A comparison between the four corner points, D1-D4, and the three central points, D5-D7, reveals that the magnitude of displacement is smaller for the former than for the latter. Comprehensive analysis of the Z-directional displacement of points D1-D7 demonstrates that points closer to the center of the soil surface experience greater displacement in the Z direction. This phenomenon arises from non-uniform soil shrinkage during the drying process, primarily due to uneven moisture distribution, leading to distortion and deformation of both the soil surface and interior. As illustrated in Fig 9, the upper portion of the soil sample undergoes more significant evaporation than the lower part due to heat-induced moisture loss. This distortion causes the soil surface to contract inward, resulting in the generation of horizontal cohesion forces. In stress analysis, this manifests as tensile stress in the horizontal direction within the soil, with higher levels observed near the central area. Additionally, the distortion effect in the vertical direction leads to increased shrinkage of the soil surface near the central area, resulting in heightened stress in the vertical direction. Consequently, "concave" areas experience downward stress in the vertical direction, with higher stress levels nearer to the central area. Boundary constraints in the edge region of the soil sample inhibit horizontal movement, leading to reduced horizontal stress at the edges, which mitigates horizontal soil contraction. Due to the constraints on the edges, greater compressive forces develop in the vertical direction, resulting in stress concentration in the edge region.

To compare the shrinkage displacement between red clay and expansive soil, soil samples C3 and E3 were selected, with a moisture content ranging from 34% to 30% for analysis. In the X direction, among the 7 characteristic points (D1 to D7), D1 exhibits the highest displacement change, measuring 2.173mm for red clay and 3.112mm for expansive soil. In the Y direction, D2 shows the maximum displacement change on the surface of both red clay and expansive soil, measuring 0.508mm and 0.684mm, respectively. In the Z direction, D5 exhibits the greatest displacement change among the seven points for both red clay and expansive soil, with a displacement change is 0.612mm for red clay and 1.236mm for expansive soil. These results indicate that when the moisture content changes similarly in both soil types, expansive soil experiences larger displacement changes in the X, Y, and Z directions compared to red clay. The difference in shrinkage between expansive soil and red clay during drying can be attributed to the particle shape and pore structure of the two soils. Red clay particles primarily exist in granular or fine-grained form, which may result in less significant interlayer water evaporation during drying and, consequently, a smaller degree of shrinkage. On the other hand, expansive soil possesses a larger pore structure compared to red clay, enabling easier moisture evaporation during drying and contributing to greater shrinkage. Figs 7 and 8 also illustrate the decrease in moisture content from 34% to 30% for both red clay and expansive soil, with expansive soil requiring less time for this process compared to red clay.

### Anisotropic shrinkage rate of soil samples

**Fig 10:**
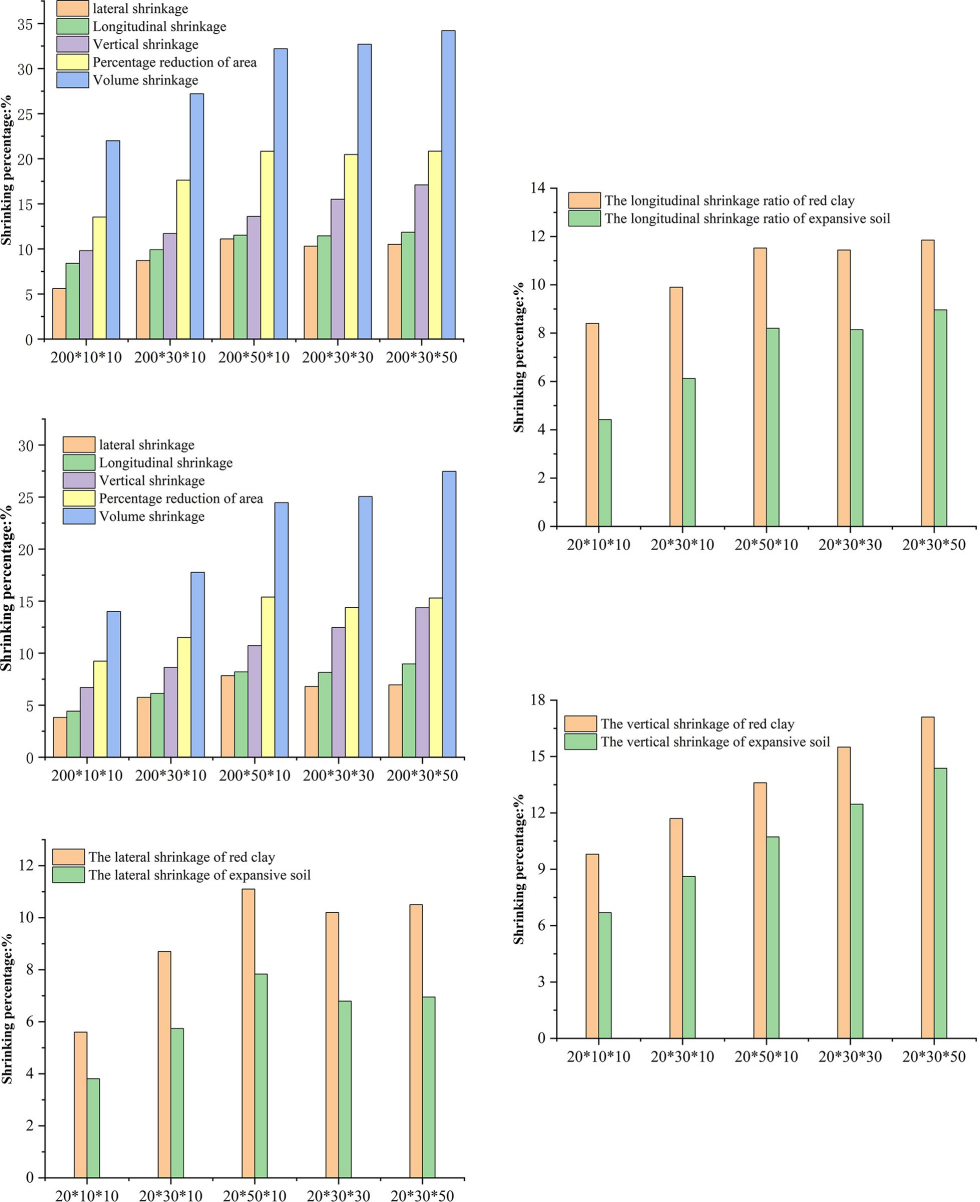
Anisotropic shrinkage of expansive soil and red clay. a. shrinkage of red clay. b. shrinkage of expansive soil. c. Lateral shrinkage comparison. d. Longitudinal shrinkage comparison. e. Vertical shrinkage comparison.

The average shrinkage values along the X, Y, and Z directions of the soil sample were calculated. This calculation was followed by the determination of the lateral, longitudinal, and vertical shrinkage rates. Subsequently, the final length and width of the soil sample were derived based on the lateral and longitudinal shrinkage rates, leading to the determination of the final surface area. From this information, the surface area shrinkage rate was calculated. Finally, the final volume of the soil sample was computed using the area shrinkage rate and vertical shrinkage rate to derive the volume shrinkage rate of the soil sample. The shrinkage rate results for both types of soil are depicted in Fig 10.

Fig 10A and 10B illustrate the shrinkage rates of two types of soil sample, focusing on the horizontal, vertical, vertical, area, and volume aspects. The results reveal a consistent pattern across different soil sample sizes: the highest shrinkage occurs in the vertical direction, followed by another vertical direction, with the least shrinkage is observed in the horizontal direction. For example, in the case of red clay sample C1, the transverse, longitudinal, and vertical shrinkage rates are 5.60%, 8.40%, and 9.80%, respectively. The vertical shrinkage rate exceeds the transverse and longitudinal rates by 1.40% and 4.20%, respectively. This indicates the presence of anisotropic shrinkage during the drying process. Wei et al. [29]. conducted drying shrinkage tests on cylindrical soil samples and found anisotropic radial and axial shrinkage, with the axial rate exceeding the radial. Similarly, V. Y. Chertkov et al. [30]. observed anisotropic shrinkage in soil samples across different directions during drying shrinkage tests.

Upon comparing soil samples with uniform thickness and length but varying widths, it is evident that the shrinkage rate is influenced by the sample’s width. In the case of red clay, samples C1, C2, and C3 exhibit lateral shrinkage rates of 5.60%, 8.70%, and 11.10%, respectively. This clearly demonstrates that as the sample width increases, so does the lateral shrinkage rate. Similarly, the longitudinal and vertical shrinkage rates of red clay samples C1, C2, and C3 follow the same tend. A comprehensive analysis concludes that, with a constant thickness and length, the lateral, longitudinal, and vertical shrinkage rates of soil samples all increase with an increasing sample width.

Furthermore, when the soil sample width and length remain constant while the thickness varies, the lateral, longitudinal, and vertical shrinkage rates of both soil types also increase with thicker samples. For example, in red clay, the lateral shrinkage rates for samples C2, C4, and C5 are 8.70%, 10.20%, and 10.50% respectively, indicating an upward trend as sample thickness increases. Similarly, the longitudinal shrinkage rates for samples C2, C4, and C5 are 9.90%, 11.44%, and 11.85%, respectively, showing a consistent increase with greater thickness. Additionally, the vertical shrinkage rates for samples C2, C4, and C5 are 11.70%, 15.50%, and 17.10%, respectively, indicating an increasing vertical shrinkage rate as the thickness becomes greater.

Fig 10C–10E provide insights into the shrinkage characteristics of both E1-E5 expansive soil samples and C1-C5 red clay samples when their width, length, and thickness are held constant. Notably, the three-dimensional (X, Y, Z) shrinkage, area shrinkage, and volume shrinkage of the expansive soil samples are smaller than those of the red clay samples. This discrepancy can be attributed to the initial moisture content of the two soil types, which were 35.58% for the expansive soil and 51.23% for the red clay in this experiment. The shrinkage deformation of the soil samples is influenced by their initial moisture content, with higher initial moisture content resulting in greater shrinkage. This observation aligns with the findings of Tang, Zhang, and Zhao [31, 32], who established a correlation between initial moisture content and shrinkage deformation in their research. The experimental results presented in this article further support this relationship.

### Soil surface strain

**Fig 11:**
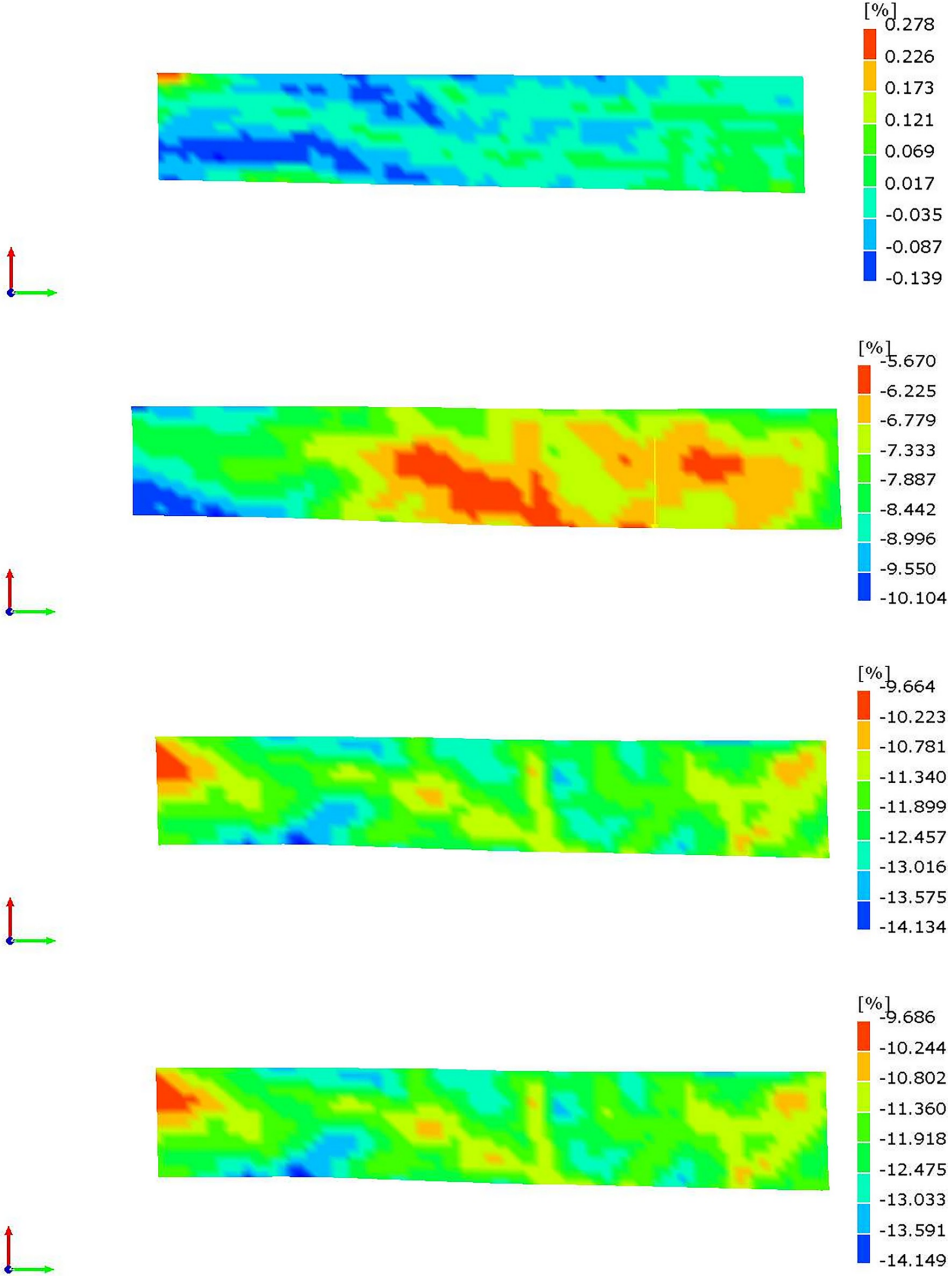
Maximum principal strain cloud of red clay. a.10min. b.240min. c.520min. d.750min.

**Fig 12:**
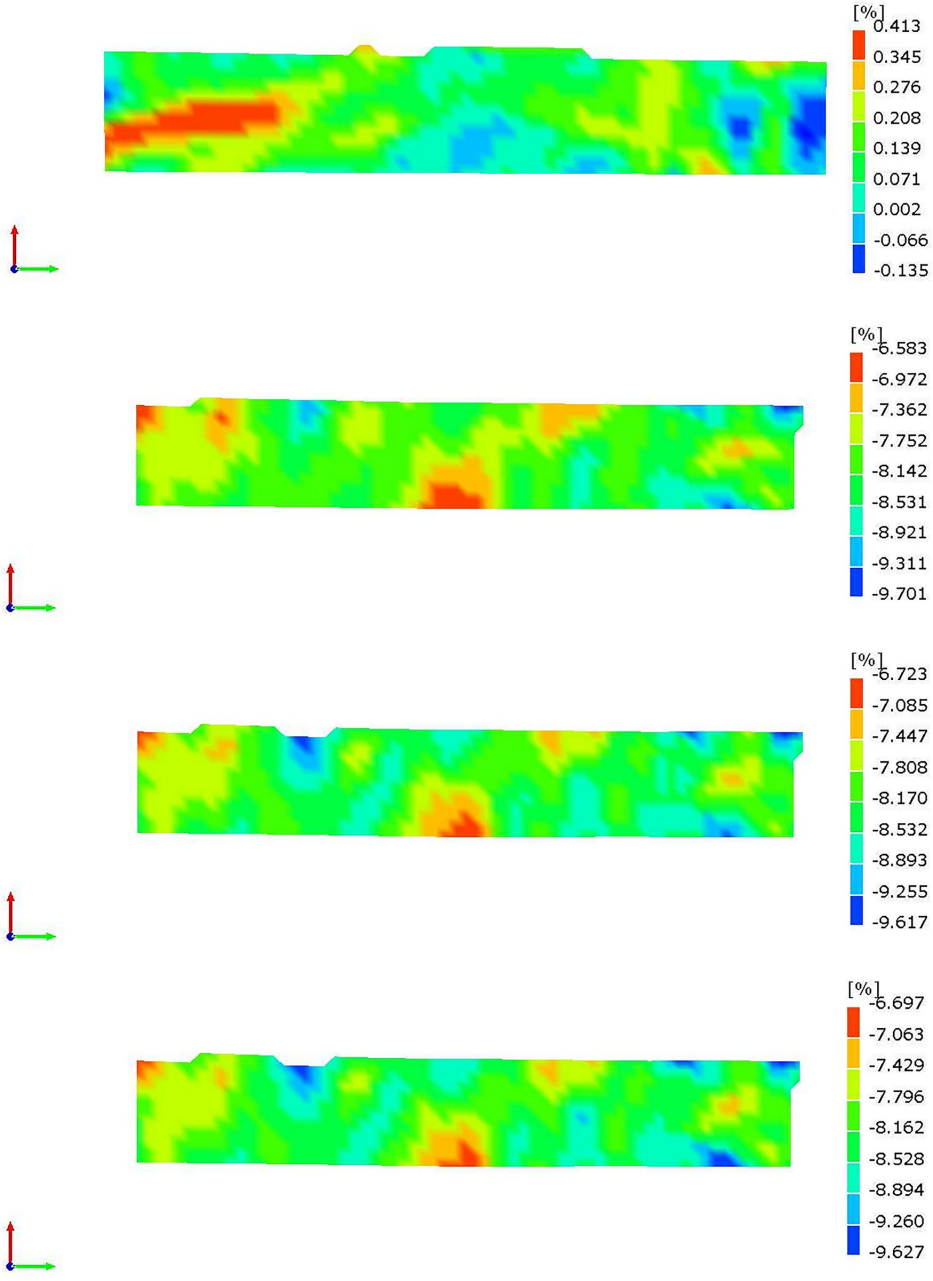
Maximum principal strain cloud of expansive soil. a.10min. b.350min. c.630min. d.960min.

When an object is simultaneously, it undergoes strain in all directions. The magnitude of strain increases with the applied force, and the highest strain reached is known as the maximum principal strain. In this experiment, the XTDIC system was utilized to monitor the evolution of the maximum principal strain in soil samples during the drying process. Figs 11 and 12 present cloud maps that depict the distribution of the maximum principal strain for soil samples C3 and E3 at distinct time intervals.

Fig 11 analysis reveals the initial occurrence of both tensile and compressive strains on the soil surface for a duration of 10 minutes. However, the principal strain values are relatively small, with a maximum tensile strain of 0.278% and a maximum compressive strain of 0.139%. After 240 minutes, the tensile strain diminishes, while the compressive strain gradually increases from the center of the soil surface towards the surrounding areas. The maximum compressive strain in the surrounding areas reaches 10.104%, while at the soil sample center, it is only 5.670% From 520 to 750 minutes, the majority of compressive strains on the soil surface vary from 11.340% to 13.016%. It’s crucial to note that the non-uniform distribution of surface strain in the soil sample may be the primary cause of its anisotropy in shrinkage. Similar conclusions were reached by Tang et al. [33] in their study on the shrinkage cracking of soil samples and suggested that there are shrinkage centers in each crack area of the soil samples.

By comparing the strain cloud maps in Figs 11 and 12, it’s evident that the surface strain distribution and development pattern of the expansive soil sample resemble those of the red clay sample. This indicates an uneven surface strain distribution of the soil sample. However, after conducting two soil tests, it is apparent that the surface compressive strain of the expansive soil is smaller compared to that of the red clay. This indirectly suggests that the shrinkage deformation of the expansive soil is less than that of the red clay. This finding validates the relationship between higher initial moisture content and greater drying deformation of the soil sample. Furthermore, Noémie Prime et al. [34] discovered that the ratio between the maximum and minimum strain directions of layered rocks is approximately 2.19, providing additional support for the concept of anisotropy in soil shrinkage based on strain analysis.

### Mechanism of drying shrinkage

**Fig 13:**
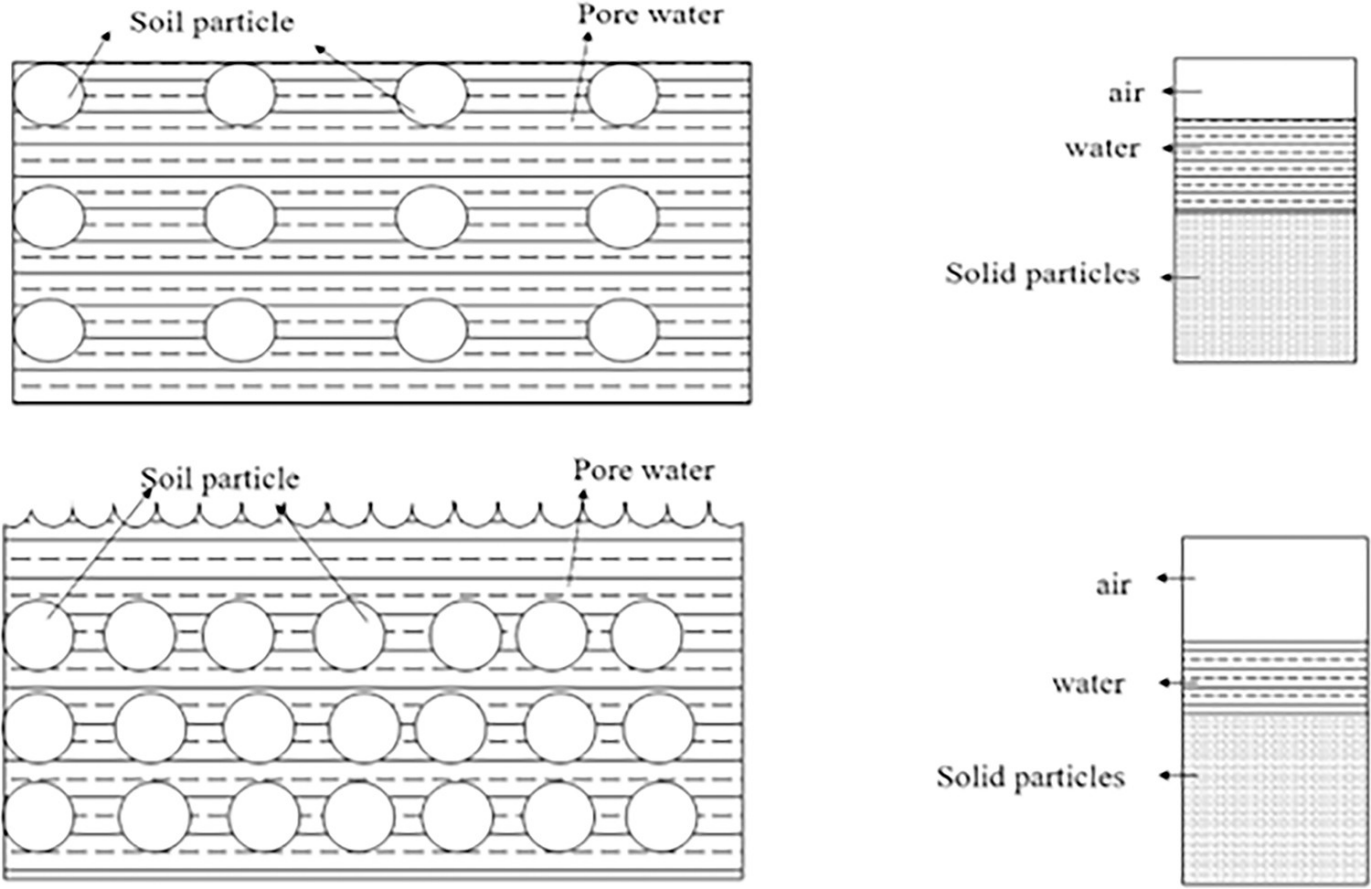
Internal particle distribution and three-phase diagram of soil at each stage of soil mass. a. Initial particle distribution, pore size and three-phase diagram of soil. b. Particle distribution, pore size and three-phase diagram of soil after drying for a certain period of time.

**Fig 14:**
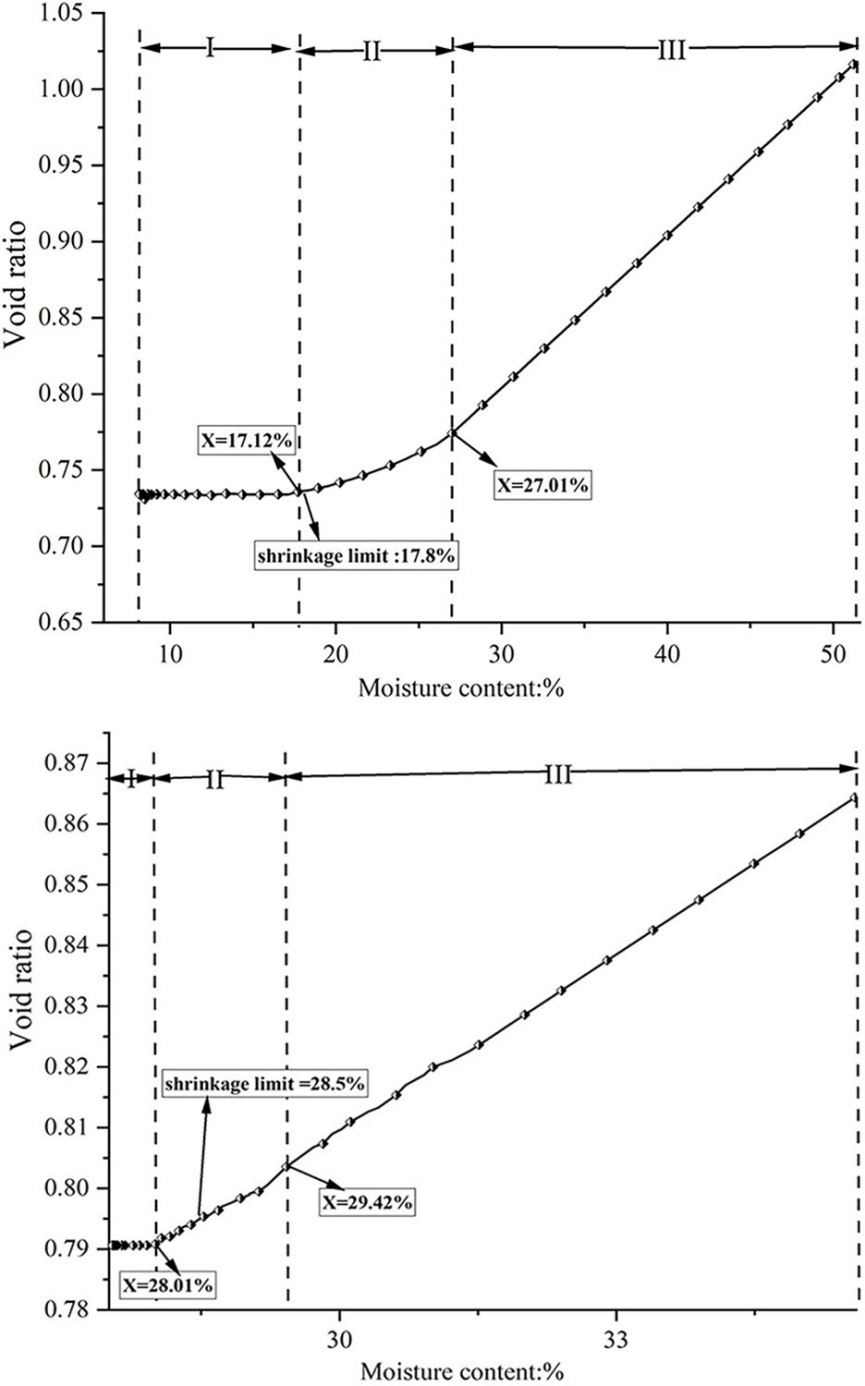
The pore ratio of soil samples varies with moisture content. a. red clay. b. expansive soil.

During the drying process, the soil sample undergoes volume shrinkage as a result of moisture loss, while the number of particles remains constant, leading to increased compactness of the soil sample [35]. Soil can be considered as a three-phase system, consisting of solid particles, water, and gas phases [36]. Initially, the soil sample contains a high moisture content, with free water occupying the pores within (Fig 13A). As the temperature of the soil surface increases during drying, the internal soil moisture decreases relative to the surrounding air, causing water to be lost from the internal pores, This leads to shrinkage of the pore shrinkage between soil particles due to capillary water pressure and surface tension., resulting in a decrease in water volume within the soil (Fig 13B) and consequently causing soil sample to shrink. Fig 14 illustrates the relationship between porosity and water content for both soil types during the experiment. The ’porosity-water content’ curves for both soils exhibit three stages: an initial linear decrease in porosity with decreasing water content, followed by concave curves as the porosity decrease rate slows down at specific water content levels. Once the soil sample moisture content reaches the shrinkage limit, the ’porosity-water content’ curve stabilizes. Figs 7 and 8 demonstrate that red clay and expansive soil reach stable displacement in the X and Y directions at moisture contents of 17.72% and 29.53%, respectively, which are close to their shrinkage limits.

Through a comprehensive analysis of Figs 7, Fig 8, and 14, the relationship between variations in soil pore ratio and development of displacement at different points on the soil surface becomes evident. Initially, as pore water fills the pores of the soil sample, volume shrinkage occurs linearly due to the loss of pore water. Upon reaching a specific moisture content, the pores are not able to fully retain water, allowing air to enter, referred to as the ’inlet point’ by Tang [36]. At this stage, the reduction in pore water results in a thinner adsorption hydration film between the soil particles, increasing particle bonding and the resistance of the soil to deformation. This leads to the ’pore ratio water content’ curve adopting a concave shape, resulting in a slower rate of displacement and entering a concave curve stage. As the moisture content of the soil decreases towards the shrinkage limit, the amount of water between soil particles diminishes, causing the particles to come into close contact. Consequently, soil deformation due to water loss ceases, maintaining a stable porosity of the soil sample, as reflected in the stabilization of the ’porosity moisture content’ curve. Consistency can be observed between the ’porosity water content’ curve of soil samples in this article and those presented by Tang [36]. and V Y Chertkov [37]. Furthermore, Wang [38]. compared the shrinkage curves of four soil types (sand, silt, clay, and soft clay) during drying, aligning with the three stages defined in this study.

The drying and shrinkage mechanisms of soil with swell-shrink characteristics are complex. Scholars have observed that as the relative humidity of the soil sample drops below 40%-50%, capillary water is no longer present, and the corresponding capillary water pressure dissipates. Instead, soil particles establish connections through a bound water film. As the pores between the soil particles became filled with bound water, the shrinkage of the soil becomes more refined, influenced by the thickness of the diffusion layer. In this stage, capillary water and surface tension play less dominant roles [39]. Furthermore it is important to note that achieving a uniformly structured internal soil sample during dry shrinkage tests poses challenges. This challenge is particularly pronounced when there are density variations across different sections of the soil sample, which can potentially lead to variations in shrinkage rates during the drying process [40–42].

## Conclusion

This study utilized the Digital Image Correlation (DIC) technique to conduct comprehensive drying-shrinkage tests on representative soils exhibiting swell-shrink characteristics, specifically Hunan red clay and Guangxi expansive soil. in various sample sizes. The investigation focused on capturing the development patterns of surface displacement and strain throughout the drying process, while also conducting an in-depth analysis of the anisotropic shrinkage and size effects observed in the soil specimens. The study findings can be summarized as follows:

(a). The progression of surface displacement in soils with swell-shrink characteristics during the drying process can be categorized into two distinct stages: initial linear growth and displacement stabilization along the X and Y directions, followed by surface uplift, vertical shrinkage, and eventual shrinkage stabilization along the Z direction.

(b). Soils exhibiting swell-shrink characteristics manifest anisotropic shrinkage tendencies during the drying phase, with the highest vertical shrinkage rate, followed by the longitudinal shrinkage rate, and the lowest transverse shrinkage rate. This observation underscores the non-uniform deformation experienced by these soils during the drying process.

(c). The shrinkage rate of soils displaying swell-shrink characteristics is influenced by the dimensions of the soil sample. Specifically, when the thickness of the soil sample matches its length, the triaxial shrinkage rate increases with expanding width. Conversely, when the width aligns with the length, the triaxial shrinkage rate escalates with thicker specimens. These findings contribute vital insights for a deeper comprehension of the size effect pertaining to soils exhibiting swell-shrink characteristics.

(d). The evolution of surface strain in soils demonstrating swell-shrink characteristics during the drying and shrinkage process follows a discernible pattern. In the initial stage, tensile strain dominates the soil surface, while as the soil continues to shrink, compressive strain becomes the prevailing factor. Variations in strain across different positions and time intervals result in an irregular and anisotropic shrinkage behavior within the soil samples.

## Supporting information

S1 Data Minimal data set file. The data in the S1 Data cover the most essential data of this manuscript, which include the information embodied in Figs 7, 8, 10, and 14 of the manuscript.
(XLSX)

S2 Data Minimal data set table. All of the data in the S2 Data cover the most essential data for this manuscript and are categorized into sections.
(PDF)
