# Supplementary material for: Study on the dry shrinkage characteristics and size effect of swell-shrink characteristic soil
Source: PLoS One. 2024 Aug 5;19(8):e0307679. doi: 10.1371/journal.pone.0307679 (PMC11299803; doi:10.1371/journal.pone.0307679)
Supplement: S2 Data — All of the data in the S2 Data cover the most essential data for this manuscript and are categorized into sections. (PDF) [file pone.0307679.s002.pdf]

All the data in the following tables cover the most basic data of this manuscript, and the following is a detailed description of the tables

1) Table A provides the data from Fig7a, specifically representing displacement of characteristic points in the X-direction of red clay soil samples and changes in moisture content of soil samples with drying time.
2) Table B provides the data from Fig7b, specifically representing displacement of characteristic points in the Y-direction of red clay soil samples and changes in moisture content of soil samples with drying time.
3) Table C provides the data from Fig7c, specifically representing displacement of characteristic points in the Z-direction of red clay soil samples and changes in moisture content of soil samples with drying time.
4) Table D provides the data from Fig8a, specifically the displacements of the characteristic points of the expansive soil samples in the X-direction and the changes in the moisture content of the soil samples as a function of drying time.
5) Table E provides the data from Fig8b, specifically the displacements of the characteristic points of the expansive soil samples in the Y-direction and the changes in the moisture content of the soil samples as a function of drying time.
6) Table F provides the data from Fig8c, specifically the displacements of the characteristic points of the expansive soil samples in the Z-direction and the changes in the moisture content of the soil samples as a function of drying time.
7) Table G provides the data from Fig10a, specifically the secretary of isotropic shrinkage of red clay soil samples.

8) Table H provides the data from Fig10b, specifically the secretary of isotropic shrinkage on expansive soil samples.

9) Table I provides the data from Fig10c, specifically the comparison of lateral shrinkage between red clay and expansive soil.

10) Table J provides the data from Fig10d, specifically the comparison of longitudinal shrinkage between red clay and expansive soil.

11) Table K provides the data from Fig10e, specifically the comparison of vertical shrinkage between red clay and expansive soil.

12) Table L provides the data from Fig13a, specifically the porosity of red clay soil samples varies with moisture content.

13) Table M provides the data from Fig13b, specifically the porosity from expansive soils varies with moisture content.

Table A Displacement of characteristic points in the X-direction of red clay soil samples and changes in moisture content of soil samples with drying time.

t	D1	D2	D3	D4	D5	D6	D7	W
min	mm	mm	mm	mm	mm	mm	mm	%
0	0	0	0	0	0	0	0	51.23
10.05	0.13258	0.14736	0.01371	0.04522	0.03374	0.06793	0.02551	50.81
20.08333	0.4716	0.44895	0.06713	0.1227	0.0908	0.20034	0.09621	50.387
30.11667	0.7358	0.70748	0.00467	0.09811	0.12141	0.3029	0.07152	49.967
40.16667	1.00996	1.07297	-0.14888	0.04028	0.11839	0.40764	0.03138	49.067
50.2	1.29357	1.45491	-0.26954	0.0156	0.14468	0.51121	-0.00885	48.167
60.25	1.47779	1.50803	-0.47279	-0.05803	0.18158	0.53269	-0.03033	47.277
70.28333	1.6719	1.61105	-0.6669	-0.16105	0.23519	0.52219	-0.01983	46.387
80.33333	1.98339	1.8729	-0.97839	-0.4229	0.1754	0.71555	-0.21319	45.497
90.36667	2.19075	2.0499	-1.18575	-0.5999	0.20084	0.78344	-0.28108	44.607
100.4167	2.45646	2.25615	-1.45146	-0.80615	0.20437	0.88663	-0.38427	43.697
110.4667	2.67289	2.47708	-1.66789	-1.02708	0.21158	0.95497	-0.45261	42.777
120.5	2.83247	2.66783	-1.82747	-1.21783	0.22666	1.00676	-0.5044	41.857
130.55	3.18911	2.93729	-2.18411	-1.48729	0.21449	1.2032	-0.70084	40.937

140.5833	3.40483	3.18276	-2.39983	-1.73276	0.22489	1.3163	-0.81394	40.017
150.6333	3.65746	3.43386	-2.65246	-1.98386	0.22851	1.43501	-0.93265	39.087
160.6667	3.89868	3.7066	-2.89368	-2.2566	0.19478	1.57811	-1.07575	38.167
170.7167	4.23104	3.98226	-3.22604	-2.53226	0.17624	1.72787	-1.22551	37.237
180.75	4.51831	4.23331	-3.51331	-2.78331	0.12513	1.90198	-1.39962	36.307
190.8	4.83397	4.51565	-3.82897	-3.06565	0.05199	2.09565	-1.59329	35.377
200.8333	5.16601	4.80999	-4.16101	-3.35999	-0.02151	2.28894	-1.78658	34.447
210.8833	5.4021	5.03563	-4.3971	-3.58563	-0.07813	2.39438	-1.89202	33.517
220.9167	5.76428	5.32649	-4.75928	-3.87649	-0.12309	2.62337	-2.12101	32.587
230.9667	6.03212	5.61334	-5.02712	-4.16334	-0.1939	2.79454	-2.29218	31.657
241	6.30138	5.85314	-5.29638	-4.40314	-0.23411	2.94478	-2.44242	30.727
251.05	6.51332	6.07029	-5.50832	-4.62029	-0.24082	3.04625	-2.54389	29.797
261.0833	6.70061	6.40139	-5.69561	-4.95139	-0.27124	3.20329	-2.70093	28.867
271.1167	6.90785	6.61421	-5.90285	-5.16421	-0.27352	3.31383	-2.81147	27.937
281.1667	7.09815	6.80069	-6.09315	-5.35069	-0.26059	3.40804	-2.90568	27.007
291.2	7.31609	7.0096	-6.31109	-5.5596	-0.27348	3.52371	-3.02135	26.077
301.25	7.57099	7.20577	-6.56599	-5.75577	-0.30123	3.65412	-3.15176	25.147
311.2833	7.81075	7.40478	-6.80575	-5.95478	-0.32264	3.79229	-3.28993	24.217
321.3333	8.00542	7.64943	-7.00042	-6.19943	-0.35022	3.92153	-3.41917	23.287
331.3667	8.20564	7.84899	-7.20064	-6.39899	-0.34493	4.00956	-3.5072	22.357
341.4167	8.40321	8.04709	-7.39821	-6.59709	-0.3444	4.1103	-3.60794	21.637
351.45	8.60152	8.24815	-7.59652	-6.79815	-0.35455	4.2243	-3.72194	20.937
361.5	8.79541	8.45632	-7.79041	-7.00632	-0.37274	4.33909	-3.83673	20.247
371.5333	8.89183	8.56938	-7.88683	-7.11938	-0.35129	4.35793	-3.85557	19.587
381.5833	9.06354	8.81733	-8.05854	-7.36733	-0.36163	4.51763	-4.01527	18.947
391.6167	9.11862	8.95245	-8.11362	-7.50245	-0.32902	4.57276	-4.0704	18.327
401.6667	9.11117	9.04151	-8.10617	-7.59151	-0.28785	4.58832	-4.08596	17.717
411.7	9.10611	9.08994	-8.10111	-7.63994	-0.24098	4.59129	-4.08893	17.117
421.7333	9.09162	9.10132	-8.08662	-7.65132	-0.20543	4.58208	-4.07972	16.537
431.7833	9.08262	9.11276	-8.07762	-7.66276	-0.19001	4.57815	-4.07579	15.977
441.8167	9.05659	9.10477	-8.05159	-7.65477	-0.15596	4.55242	-4.05006	15.427
451.85	8.98235	9.01115	-7.97735	-7.56115	-0.13532	4.50871	-4.00635	14.897
461.9	9.01317	9.05438	-8.00817	-7.60438	-0.12308	4.47994	-3.97758	14.377
471.9333	9.02095	9.0935	-8.01595	-7.6435	-0.11419	4.52729	-4.02493	13.877
481.9667	8.92237	9.06851	-7.91737	-7.61851	-0.10136	4.45392	-3.95156	13.397
492.0167	8.9919	9.07412	-7.9869	-7.62412	-0.07942	4.50015	-3.99779	12.937
502.05	8.97778	9.05743	-7.97278	-7.60743	-0.06365	4.48515	-3.98279	12.487
512.1	8.97068	9.05241	-7.96568	-7.60241	-0.05578	4.47738	-3.97502	12.057
522.1333	8.97512	9.00303	-7.97012	-7.55303	-0.04346	4.48244	-3.98008	11.657
532.1667	8.98162	9.06916	-7.97662	-7.61916	-0.06512	4.48818	-3.98582	11.277
542.2167	8.95735	9.00112	-7.95235	-7.55112	-0.02605	4.45868	-3.95632	10.907
552.25	8.9458	9.03043	-7.9408	-7.58043	-0.02427	4.44459	-3.94223	10.557
562.3	8.93977	9.01732	-7.93477	-7.56732	-0.03287	4.45157	-3.94921	10.237
572.3333	8.94466	9.02763	-7.93966	-7.57763	-0.02782	4.44847	-3.94611	9.937

582.3667	8.95365	9.03288	-7.94865	-7.58288	-0.02935	4.45447	-3.95211	9.657
592.4167	8.94936	9.0371	-7.94436	-7.5871	-0.03121	4.45366	-3.9513	9.407
602.45	8.96084	9.0324	-7.95584	-7.5824	-0.01035	4.4497	-3.94734	9.197
612.5	8.9481	9.03481	-7.9431	-7.58481	-0.02439	4.44924	-3.94688	9.017
622.5333	8.94749	9.035	-7.94249	-7.585	-0.02631	4.45187	-3.94951	8.877
632.5833	8.9242	9.01594	-7.9192	-7.56594	-0.02637	4.43546	-3.9331	8.777
642.6167	8.92127	9.04056	-7.91627	-7.59056	-1.04E-02	4.41027	-3.90791	8.677
652.65	8.96486	9.02463	-7.95986	-7.57463	-0.03717	4.46475	-3.96239	8.577
662.7	8.96219	9.04466	-7.95719	-7.59466	-0.02661	4.45718	-3.95482	8.477
672.7333	8.96922	9.04515	-7.96422	-7.59515	-0.0141	4.45594	-3.95358	8.427
682.7833	8.95626	9.04139	-7.95126	-7.59139	-0.02487	4.45437	-3.95201	8.377
692.8167	8.97489	9.06359	-7.96989	-7.61359	-0.02617	4.46386	-3.9615	8.327
692.8667	8.81306	8.88876	-7.80806	-7.43876	1.83E-04	4.40773	-3.90537	8.287
702.9	8.87929	9.05867	-7.87429	-7.60867	-0.00188	4.44611	-3.94375	8.247
712.9333	8.97232	9.06417	-7.96732	-7.61417	-0.01868	4.48184	-3.97948	8.207
722.9833	8.92372	8.99604	-7.91872	-7.54604	-0.02827	4.43679	-3.93443	8.177
733.0167	8.96541	9.00494	-7.96041	-7.55494	-0.02928	4.45266	-3.9503	8.157
743.0333	8.76701	8.97012	-7.76201	-7.52012	0.06575	4.42052	-3.91816	8.147

Table B Displacement of characteristic points in the Y-direction of red clay soil samples and changes in moisture content of soil samples with drying time.

t	D1	D2	D3	D4	D5	D6	D7	W
min	mm	mm	mm	mm	mm	mm	mm	%
0	0	0	0	0	0	0	0	51.23
10.05	0.10273	0.05423	0.07285	0.04331	0.09388	0.05508	0.03388	50.81
20.08333	-0.12742	0.05857	-0.09752	0.04765	0.05243	0.01363	-0.00757	50.387
30.11667	-0.21878	0.1568	-0.18888	0.14588	-0.00688	-0.04568	-0.06688	49.967
40.16667	-0.30125	0.2568	-0.27136	0.24588	-0.04956	-0.08836	-0.10956	49.067
50.2	-0.41258	0.3568	-0.38268	0.34588	-0.06973	-0.10853	-0.12973	48.167
60.25	-0.51236	0.4658	-0.48246	0.45488	-0.07095	-0.10975	-0.13095	47.277
70.28333	-0.6258	0.5624	-0.5959	0.55148	-0.07392	-0.11272	-0.13392	46.387
80.33333	-0.71457	0.64123	-0.68467	0.63031	-0.16971	-0.20851	-0.22971	45.497
90.36667	-0.80235	0.76258	-0.77245	0.75166	-0.17383	-0.21263	-0.23383	44.607
100.4167	-0.91235	0.85985	-0.88245	0.84893	-0.24115	-0.27995	-0.30115	43.697
110.4667	-1.02895	0.95124	-0.99905	0.94031	-0.25087	-0.28967	-0.31087	42.777
120.5	-1.1235	1.05995	-1.0936	1.04903	-0.32801	-0.36681	-0.38801	41.857
130.55	-1.23552	1.14592	-1.20562	1.135	-0.35993	-0.39873	-0.41993	40.937
140.5833	-1.35695	1.23599	-1.32706	1.22507	-0.47154	-0.51034	-0.53154	40.017
150.6333	-1.4569	1.35126	-1.427	1.34034	-0.54728	-0.58608	-0.60728	39.087
160.6667	-1.56288	1.45985	-1.53298	1.44893	-0.61058	-0.64938	-0.67058	38.167
170.7167	-1.6528	1.65892	-1.6229	1.648	-0.66051	-0.69931	-0.72051	37.237
180.75	-1.75922	1.75269	-1.72932	1.74177	-0.68925	-0.72805	-0.74925	36.307

190.8	-1.8628	1.86952	-1.8329	1.8586	-0.78241	-0.82121	-0.84241	35.377
200.8333	-1.9658	1.95237	-1.9359	1.94144	-0.81735	-0.85615	-0.87735	34.447
210.8833	-2.0536	2.0255	-2.0237	2.01458	-0.92207	-0.96087	-0.98207	33.517
220.9167	-2.15088	2.1336	-2.12098	2.12267	-0.94978	-0.98858	-1.00978	32.587
230.9667	-2.23698	2.24953	-2.20708	2.23861	-1.02513	-1.06393	-1.08513	31.657
241	-2.44268	2.356898	-2.41278	2.3536	-1.13343	-1.17223	-1.19343	30.727
251.05	-2.53284	2.4592	-2.50294	2.44828	-1.19351	-1.23231	-1.25351	29.797
261.0833	-2.64236	2.56282	-2.61246	2.5519	-1.19139	-1.23019	-1.25139	28.867
271.1167	-2.74598	2.65865	-2.71608	2.64773	-1.2603	-1.2991	-1.3203	27.937
281.1667	-2.85282	2.746	-2.82292	2.73507	-1.31562	-1.35442	-1.37562	27.007
291.2	-2.95268	2.85236	-2.92278	2.84144	-1.36941	-1.40821	-1.42941	26.077
301.25	-3.06285	2.95236	-3.03295	2.94144	-1.40183	-1.44063	-1.46183	25.147
311.2833	-3.16582	3.05236	-3.13592	3.04144	-1.42036	-1.45916	-1.48036	24.217
321.3333	-3.26582	3.15235	-3.23592	3.14143	-1.43364	-1.47244	-1.49364	23.287
331.3667	-3.46285	3.25467	-3.43295	3.24374	-1.44299	-1.48179	-1.50299	22.357
341.4167	-3.55258	3.34265	-3.52268	3.33173	-1.46344	-1.50224	-1.52344	21.637
351.45	-3.65821	3.44569	-3.62831	3.43476	-1.47832	-1.51712	-1.53832	20.937
361.5	-3.75952	3.5628	-3.72962	3.55188	-1.48922	-1.52802	-1.54922	20.247
371.5333	-3.86892	3.65215	-3.83902	3.64122	-1.53037	-1.56917	-1.59037	19.587
381.5833	-3.92562	3.75127	-3.89573	3.74034	-1.53562	-1.57442	-1.59562	18.947
391.6167	-4.05238	3.85126	-4.02248	3.84033	-1.54093	-1.57973	-1.60093	18.327
401.6667	-4.15255	3.95982	-4.12265	3.9489	-1.54008	-1.57888	-1.60008	17.717
411.7	-4.18383	3.99548	-4.15393	3.98456	-1.53817	-1.57697	-1.59817	17.117
421.7333	-4.19244	4.01034	-4.16254	3.99942	-1.53511	-1.57391	-1.59511	16.537
431.7833	-4.19955	4.01249	-4.16965	4.00156	-1.52864	-1.56744	-1.58864	15.977
441.8167	-4.19228	4.01271	-4.16238	4.00178	-1.53405	-1.57285	-1.59405	15.427
451.85	-4.22904	4.01724	-4.19914	4.00632	-1.53526	-1.57406	-1.59526	14.897
461.9	-4.22697	4.02403	-4.19707	4.01311	-1.53461	-1.57341	-1.59461	14.377
471.9333	-4.27325	4.02659	-4.24335	4.01566	-1.53294	-1.57174	-1.59294	13.877
481.9667	-4.28976	4.02735	-4.25986	4.01643	-1.53381	-1.57261	-1.59381	13.397
492.0167	-4.29237	4.02835	-4.26247	4.01743	-1.54142	-1.58022	-1.60142	12.937
502.05	-4.29498	4.03733	-4.26508	4.02641	-1.5353	-1.5741	-1.5953	12.487
512.1	-4.29996	4.04033	-4.27006	4.02941	-1.53389	-1.57269	-1.59389	12.057
522.1333	-4.30015	4.0466	-4.27025	4.03568	-1.538	-1.5768	-1.598	11.657
532.1667	-4.30905	4.04024	-4.27915	4.02932	-1.53985	-1.57865	-1.59985	11.277
542.2167	-4.30589	4.04271	-4.27599	4.03179	-1.53684	-1.57564	-1.59684	10.907
552.25	-4.30078	4.0402	-4.27089	4.02928	-1.5325	-1.5713	-1.5925	10.557
562.3	-4.29386	4.0402	-4.26396	4.02928	-1.52602	-1.56482	-1.58602	10.237
572.3333	-4.29938	4.0402	-4.26948	4.02928	-1.53098	-1.56978	-1.59098	9.937
582.3667	-4.29061	4.04271	-4.26071	4.03179	-1.52698	-1.56578	-1.58698	9.657
592.4167	-4.29915	4.0402	-4.26925	4.02928	-1.53212	-1.57092	-1.59212	9.407
602.45	-4.29796	4.04271	-4.26806	4.03179	-1.53183	-1.57063	-1.59183	9.197
612.5	-4.30168	4.04693	-4.27178	4.03601	-1.53654	-1.57534	-1.59654	9.017
622.5333	-4.30842	4.04363	-4.27852	4.03271	-1.5311	-1.5699	-1.5911	8.877

632.5833	-4.30011	4.04561	-4.27021	4.03469	-1.53528	-1.57408	-1.59528	8.777
642.6167	-4.30452	4.04294	-4.27462	4.03202	-1.53911	-1.57791	-1.59911	8.677
652.65	-4.30514	4.04272	-4.27524	4.0318	-1.53317	-1.57197	-1.59317	8.577
662.7	-4.30572	4.04597	-4.27582	4.03505	-1.53966	-1.57846	-1.59966	8.477
672.7333	-4.31662	4.04305	-4.28672	4.03213	-1.54047	-1.57927	-1.60047	8.427
682.7833	-4.31889	4.04034	-4.28899	4.02942	-1.52635	-1.56515	-1.58635	8.377
692.8167	-4.31054	4.04017	-4.28064	4.02925	-1.53612	-1.57492	-1.59612	8.327
692.8667	-4.31464	4.03958	-4.28474	4.02866	-1.54019	-1.57899	-1.60019	8.287
702.9	-4.31925	4.04293	-4.28935	4.03201	-1.53591	-1.57471	-1.59591	8.247
712.9333	-4.31231	4.04038	-4.28241	4.02946	-1.53454	-1.57334	-1.59454	8.207
722.9833	-4.31816	4.04271	-4.28826	4.03179	-1.54013	-1.57893	-1.60013	8.177
733.0167	-4.31821	4.03963	-4.28831	4.02871	-1.54124	-1.58004	-1.60124	8.157
743.0333	-4.31823	4.04034	-4.28833	4.02942	-1.52735	-1.56615	-1.58735	8.147

Table C Displacement of characteristic points in the Z-direction of red clay soil samples and changes in moisture content of soil samples with drying time.

t	D1	D2	D3	D4	G5	D6	D7	W
min	mm	mm	mm	mm	mm	mm	mm	%
0	0	0	0	0	0	0	0	51.23
10.05	0.40722	0.34249	0.1276	0.0782	0.24072	0.13425	0.1276	50.81
20.08333	0.50183	0.4493	0.26518	0.17855	0.46718	0.26093	0.26518	50.387
30.11667	0.60545	0.51582	0.3258	0.26387	0.68261	0.38006	0.38265	49.967
40.16667	0.7159	0.61568	0.40258	0.36258	0.81653	0.5224	0.52467	49.067
50.2	0.81258	0.71599	0.5128	0.46588	1.02844	1.00702	0.70959	48.167
60.25	0.91238	0.81569	0.6258	0.56268	1.22471	0.97611	0.81384	47.277
70.28333	1.02585	0.91389	0.71258	0.62585	1.31517	1.05179	0.9607	46.387
80.33333	1.13589	0.9719	0.81258	0.74268	1.41502	1.05076	1.0295	45.497
90.36667	1.2458	1.07003	0.9258	0.8628	1.51025	1.15563	1.14931	44.607
100.4167	1.32588	1.17355	1.0328	0.9628	1.56724	1.25961	1.23313	43.697
110.4667	1.4289	1.27463	1.05699	0.98295	1.60235	1.36116	1.35975	42.777
120.5	1.4589	1.31239	1.0803	1.04285	1.65286	1.46417	1.40509	41.857
130.55	1.3258	1.22445	0.739	0.63758	1.35126	1.36072	1.33812	40.937
140.5833	1.2017	1.10968	0.45192	0.3672	0.86963	0.84113	0.9343	40.017
150.6333	0.96226	0.95481	0.40024	0.23617	0.54841	0.74809	0.16309	39.087
160.6667	0.87129	0.85997	0.39779	0.19334	0.30941	0.51332	-0.08719	38.167
170.7167	0.82221	0.80166	0.30442	0.07752	0.18127	0.43587	-0.26295	37.237
180.75	0.76607	0.74593	0.18084	0.03103	0.14564	0.34163	-0.46397	36.307
190.8	0.66243	0.61594	0.16009	0.01952	-0.06912	0.13042	-0.61469	35.377
200.8333	0.5151	0.44702	0.14023	-0.0658	-0.12396	0.08379	-0.72823	34.447
210.8833	0.42185	0.35755	0.04853	-0.1258	-0.35961	-0.15332	-0.94189	33.517
220.9167	0.23801	0.19486	-0.06707	-0.15981	-0.4322	-0.23277	-1.01522	32.587
230.9667	0.13944	0.10584	-0.20227	-0.2174	-0.58735	-0.42208	-1.17387	31.657
241	0.11203	0.08263	-0.3569	-0.37434	-0.71161	-0.56716	-1.39307	30.727

251.05	-0.05317	-0.04003	-0.47284	-0.48773	-0.99982	-0.81847	-1.46623	29.797
261.0833	-0.09535	0.06445	-0.56018	-0.58864	-1.17914	-0.97739	-1.53917	28.867
271.1167	-0.27662	-0.11794	-0.72453	-0.74335	-1.22827	-1.00289	-1.72959	27.937
281.1667	-0.33246	-0.17905	-0.78094	-0.80567	-1.49057	-1.31763	-1.79936	27.007
291.2	-0.45181	-0.32468	-0.90298	-0.93576	-1.59193	-1.42886	-1.8948	26.077
301.25	-0.54739	-0.43628	-1.07159	-1.11707	-1.81451	-1.61025	-2.07094	25.147
311.2833	-0.76909	-0.62959	-1.12787	-1.15438	-1.91552	-1.73489	-2.19363	24.217
321.3333	-0.90703	-0.75184	-1.18398	-1.21849	-2.03233	-1.8475	-2.35411	23.287
331.3667	-0.94482	-0.77145	-1.33045	-1.37945	-2.39495	-2.13023	-2.4284	22.357
341.4167	-1.14659	-1.01356	-1.38412	-1.4166	-2.39733	-2.20095	-2.55416	21.637
351.45	-1.22456	-1.09912	-1.49846	-1.51687	-2.54375	-2.34914	-2.63519	20.937
361.5	-1.39578	-1.23865	-1.66995	-1.69063	-2.72636	-2.52485	-2.80756	20.247
371.5333	-1.47348	-1.33453	-1.68741	-1.70514	-2.83422	-2.61997	-2.82594	19.587
381.5833	-1.56333	-1.42115	-1.81085	-1.8186	-2.98649	-2.84018	-2.99624	18.947
391.6167	-1.74227	-1.53864	-1.868	-1.88303	-3.07922	-2.89221	-3.04377	18.327
401.6667	-1.8441	-1.69304	-1.96474	-1.97637	-3.21786	-3.04113	-3.19674	17.717
411.7	-1.95673	-1.80703	-2.02707	-2.03795	-3.34038	-3.13728	-3.49899	17.117
421.7333	-2.0972	-1.94219	-2.15966	-2.16589	-3.49794	-3.31872	-3.32197	16.537
431.7833	-2.18017	-2.01536	-2.1738	-2.18541	-3.71287	-3.50476	-3.43909	15.977
441.8167	-2.2973	-2.18475	-2.3048	-2.32799	-3.90113	-3.72229	-3.5133	15.427
451.85	-2.36863	-2.22478	-2.34136	-2.35658	-3.91333	-3.71466	-3.56769	14.897
461.9	-2.47528	-2.33933	-2.45903	-2.46771	-4.16775	-3.92006	-3.64826	14.377
471.9333	-2.56952	-2.41329	-2.49153	-2.53395	-4.14638	-3.93806	-3.75775	13.877
481.9667	-2.69072	-2.55286	-2.55536	-2.5816	-4.42709	-4.23925	-3.92037	13.397
492.0167	-2.80605	-2.69597	-2.64545	-2.6972	-4.60786	-4.39267	-4.20183	12.937
502.05	-2.90087	-2.7633	-2.70254	-2.71925	-4.91748	-4.73519	-4.38051	12.487
512.1	-3.01025	-2.85743	-2.74438	-2.76272	-5.03091	-4.83698	-4.5891	12.057
522.1333	-3.20596	-3.01543	-2.80635	-2.84548	-5.29658	-5.0817	-4.82944	11.657
532.1667	-3.28952	-3.12928	-2.89058	-2.92047	-5.527	-5.34065	-5.00575	11.277
542.2167	-3.40545	-3.26096	-3.01567	-3.02718	-5.48146	-5.30316	-5.15997	10.907
552.25	-3.54451	-3.37898	-3.23218	-3.39912	-5.59187	-5.40706	-5.2062	10.557
562.3	-3.78268	-3.64245	-3.36962	-3.48124	-5.64668	-5.50807	-5.28475	10.237
572.3333	-3.86993	-3.72075	-3.53008	-3.63307	-5.71493	-5.57025	-5.37226	9.937
582.3667	-4.07429	-3.909	-3.61495	-3.76642	-5.78971	-5.65652	-5.48037	9.657
592.4167	-4.25154	-4.10819	-3.75058	-3.86398	-5.90502	-5.78812	-5.48218	9.407
602.45	-4.29651	-4.13935	-3.86921	-3.97262	-5.93582	-5.80723	-5.52117	9.197
612.5	-4.30144	-4.13928	-3.97048	-3.95652	-5.93055	-5.84274	-5.5588	9.017
622.5333	-4.3436	-4.13698	-4.00652	-3.95133	-5.94302	-5.84848	-5.5547	8.877
632.5833	-4.3561	-4.12848	-4.00133	-3.95216	-5.95636	-5.86813	-5.57681	8.777
642.6167	-4.35362	-4.13512	-4.00216	-3.95217	-5.96722	-5.86175	-5.57618	8.677
652.65	-4.35423	-4.14524	-4.00217	-3.95247	-5.97707	-5.84923	-5.57596	8.577
662.7	-4.34891	-4.13902	-4.00247	-3.95276	-5.9708	-5.85825	-5.57061	8.477
672.7333	-4.3485	-4.14175	-4.00276	-3.95288	-6.00101	-5.86796	-5.57445	8.427
682.7833	-4.35716	-4.14988	-4.00288	-3.9529	-6.01367	-5.86808	-5.56752	8.377

692.8167	-4.35357	-4.13702	-4.0029	-3.95322	-6.00801	-5.86821	-5.56522	8.327
692.8667	-4.35902	-4.14509	-4.00322	-3.95315	-6.00361	-5.87941	-5.57835	8.287
702.9	-4.36516	-4.14576	-4.00315	-3.954	-6.00103	-5.86159	-5.57981	8.247
712.9333	-4.36724	-4.1466	-4.004	-3.95373	-6.00394	-5.86841	-5.57887	8.207
722.9833	-4.36162	-4.15369	-4.00373	-3.95366	-6.00777	-5.86847	-5.57694	8.177
733.0167	-4.36259	-4.15237	-4.00366	-3.95329	-6.00329	-5.86491	-5.58064	8.157
743.0333	-4.36124	-4.15396	-4.00329	-3.95826	-6.0091	-5.86986	-5.76971	8.147

Table D Displacement of characteristic points in the X-direction of expansive soil samples and changes in moisture content of soil samples with drying time.

t<br>min	D1<br>mm	D2<br>mm	D3<br>mm	D4<br>mm	D5<br>mm	D6<br>mm	D7<br>mm	W<br>%
0	0	0	0	0	0	0	0	35.58648
10.05	-0.00381	0.02821	0.04154	0.05476	0.0256	0.02057	0.01892	35.38767
20.08333	-0.01131	0.05648	0.02764	0.05618	0.0224	0.01411	0.03463	35.18887
30.11667	-0.0062	0.10479	0.00844	0.05508	0.04426	0.02594	0.04269	34.99006
40.16667	-0.00639	0.18804	-0.05259	-0.0179	0.08735	0.03266	0.08777	34.79125
50.2	-0.00114	0.30383	-0.23739	-0.23419	0.11508	0.05151	0.09142	34.59245
60.25	0.01538	0.45494	-0.39003	-0.44744	0.11494	0.07644	0.08517	34.49304
70.28333	0.02188	0.57073	-0.59482	-0.60178	0.13566	0.1244	0.09299	34.29423
80.33333	0.10978	0.76019	-0.769	-0.76128	0.16596	0.17636	0.06743	34.09543
90.36667	0.69718	1.1125	-0.99218	-0.9528	0.26428	0.41623	0.02239	33.89662
100.4167	1.09701	1.43351	-1.136	-1.17198	0.33066	0.57634	0.04291	33.79722
110.4667	1.35952	1.68936	-1.44048	-1.45803	0.33064	0.70165	-0.04691	33.59841
120.5	1.58178	1.91386	-1.72257	-1.79095	0.32967	0.79602	-0.19286	33.3996
130.55	1.75818	2.11013	-1.9229	-2.02819	0.31331	0.89238	-0.34831	33.3002
140.5833	1.9468	2.30745	-2.19229	-2.29301	0.29214	0.9659	-0.47359	33.10139

150.6333	2.11735	2.48	-2.42653	-2.5666	0.23541	1.02992	-0.61978	32.90258
160.6667	2.28856	2.65704	-2.69777	-2.86601	0.21172	1.1071	-0.76697	32.80318
170.7167	2.48326	2.84892	-2.89675	-3.05424	0.13176	1.18193	-0.90196	32.60437
180.75	2.65567	3.0282	-3.19895	-3.37944	0.11888	1.26881	-1.01031	32.40557
190.8	2.83122	3.21199	-3.45883	-3.64757	0.07423	1.33125	-1.15844	32.30616
200.8333	3.02003	3.38952	-3.70784	-3.87331	0.04727	1.39581	-1.26853	32.10736
210.8833	3.19704	3.57697	-3.9302	-4.129	0.0331	1.48197	-1.37775	32.00795
220.9167	3.438	3.8107	-4.17429	-4.38428	0.04808	1.59501	-1.46243	31.80915
230.9667	3.6638	4.02989	-4.33753	-4.5424	0.07604	1.70364	-1.5038	31.70974
241	3.87921	4.25849	-4.58121	-4.78217	0.08375	1.80652	-1.6059	31.51093
251.05	4.1094	4.47775	-4.77091	-4.97194	0.10026	1.91346	-1.64343	31.41153
261.0833	4.33065	4.68435	-4.98346	-5.15022	0.1002	2.01067	-1.77479	31.21272
271.1167	4.52008	4.88994	-5.17266	-5.3257	0.09282	2.09679	-1.87741	31.01392
281.1667	4.73899	5.07999	-5.3191	-5.47489	0.10418	2.19443	-1.9349	30.91451
291.2	4.94115	5.24418	-5.46588	-5.58867	0.14083	2.28131	-2.0306	30.71571
301.25	5.10155	5.39721	-5.59746	-5.71047	0.09756	2.36793	-2.11474	30.6163
311.2833	5.26953	5.53568	-5.70306	-5.8063	0.0853	2.44244	-2.19018	30.4175
321.3333	5.45069	5.65378	-5.80796	-5.89361	0.08163	2.51465	-2.28716	30.31809
331.3667	5.59186	5.7532	-5.86563	-5.9349	0.09924	2.59038	-2.35187	30.11928
341.4167	5.721	5.84317	-5.90638	-5.96118	0.04597	2.60669	-2.39228	30.01988
351.45	5.79328	5.87038	-5.95472	-5.99647	0.07057	2.6595	-2.46511	29.92048
361.5	5.87254	5.90716	-6.00938	-6.0417	0.04783	2.67644	-2.50999	29.82107

371.5333	5.90952	5.93522	-6.02098	-6.05271	0.02767	2.68602	-2.55097	29.72167
381.5833	5.9372	5.95826	-6.04467	-6.07412	0.01183	2.69071	-2.55738	29.52286
391.6167	5.95739	5.97451	-6.02179	-6.05521	-0.00939	2.69522	-2.58967	29.42346
401.6667	5.98455	5.98951	-6.0696	-6.14587	0.01141	2.69647	-2.55693	29.32406
411.7	5.9875	5.99265	-6.07599	-6.11432	-1.67E-04	2.70645	-2.60727	29.22465
421.7333	5.99257	5.99957	-6.06727	-6.11812	-0.02372	2.70317	-2.63244	29.12525
431.7833	6.01233	6.008	-6.09718	-6.11533	-0.0258	2.70764	-2.64022	29.02584
441.8167	6.02415	6.02012	-6.11881	-6.12267	-0.01718	2.71733	-2.64168	29.02584
451.85	6.02621	6.01955	-6.09927	-6.10788	-0.02862	2.71187	-2.66191	28.92644
461.9	6.04951	6.03806	-6.11467	-6.12796	-0.04108	2.71311	-2.67671	28.82704
471.9333	6.04578	6.03648	-6.11929	-6.12927	-0.03794	2.71498	-2.67351	28.72763
481.9667	6.06124	6.05697	-6.12476	-6.1506	-0.03919	2.70904	-2.68978	28.68763
492.0167	6.06352	6.05124	-6.1249	-6.13308	-0.04521	2.71946	-2.68106	28.62823
502.05	6.0826	6.06867	-6.18161	-6.15058	-0.03384	2.73023	-2.67935	28.58823
512.1	6.08198	6.07952	-6.13325	-6.13585	-0.04724	2.75077	-2.70042	28.52883
522.1333	6.08746	6.06419	-6.12039	-6.1332	-0.00162	2.73025	-2.70172	28.48883
532.1667	6.08254	6.05767	-6.15225	-6.16618	-0.04219	2.73646	-2.71	28.42942
542.2167	6.09809	6.09958	-6.15023	-6.16609	-0.05519	2.74762	-2.67839	28.38942
552.25	6.08472	6.07144	-6.1455	-6.11193	-0.05291	2.73555	-2.72051	28.33002
562.3	6.09525	6.08349	-6.14602	-6.14297	-0.05329	2.73894	-2.70827	28.30002
572.3333	6.10091	6.08456	-6.13451	-6.14092	-0.06926	2.72546	-2.68087	28.26002
582.3667	6.09912	6.08833	-6.1324	-6.14877	-0.03037	2.74156	-2.66081	28.23062

592.4167	6.10535	6.10054	-6.14056	-6.13861	-0.06455	2.72792	-2.72062	28.19062
602.45	6.09604	6.08713	-6.13427	-6.13853	-0.0617	2.72319	-2.71615	28.16062
612.5	6.11377	6.09831	-6.14708	-6.16994	-0.0403	2.72397	-2.7059	28.13121
622.5333	6.10369	6.09568	-6.12456	-6.14427	-0.06175	2.72803	-2.72106	28.10121
632.5833	6.10942	6.09484	-6.13799	-6.13762	-0.07895	2.72785	-2.73451	28.07121
642.6167	6.10056	6.09504	-6.15423	-6.15112	-0.06239	2.72778	-2.72001	28.06121
652.65	6.11327	6.104	-6.14427	-6.13659	-0.06364	2.72674	-2.71901	28.03181
662.7	6.10862	6.09953	-6.16058	-6.15344	-0.05327	2.72923	-2.7132	28.00181
672.7333	6.1044	6.10452	-6.1846	-6.1736	-0.07802	2.73661	-2.69768	27.97818
682.7833	6.12298	6.11048	-6.1472	-6.13548	-0.05643	2.73372	-2.71189	27.95181
692.8167	6.12519	6.12334	-6.16102	-6.14983	-0.00105	2.72964	-2.69552	27.91181
692.8667	6.12988	6.11723	-6.14408	-6.15377	-0.06183	2.72872	-2.70441	27.88241
702.9	6.09574	6.10427	-6.15015	-6.15636	-0.06242	2.74712	-2.72435	27.85241
712.9333	6.10999	6.09907	-6.15896	-6.1543	-0.07329	2.72462	-2.73052	27.83241
722.9833	6.12521	6.11314	-6.15623	-6.13558	-0.05671	2.73128	-2.71856	27.817
733.0167	6.1192	6.10269	-6.16521	-6.14808	-0.06818	2.72302	-2.72797	27.79241
743.0333	6.13922	6.11996	-6.1389	-6.1228	-0.06369	2.75245	-2.69777	27.76241
753.03	6.11869	6.10939	-6.15396	-6.16584	-0.07331	2.72915	-2.72175	27.72241
763.03	6.11664	6.11186	-6.15984	-6.14704	-0.07743	2.72912	-2.73943	27.693
773.03	6.12097	6.11892	-6.16751	-6.16186	-0.05543	2.73131	-2.73431	27.673
783.03	6.12018	6.11668	-6.18622	-6.16542	-0.06739	2.75247	-2.70612	27.653
793.03	6.12341	6.1132	-6.15184	-6.13201	-0.06854	2.74533	-2.67587	27.643

803.03	6.12579	6.11584	-6.14963	-6.14857	-0.06119	2.7335	-2.72016	27.6423
813.03	6.12326	6.11584	-6.16442	-6.14164	-0.06019	2.75059	-2.74756	27.633
823.03	6.13349	6.11671	-6.15603	-6.14155	-0.06383	2.73662	-2.72531	27.613
833.03	6.13129	6.11545	-6.16906	-6.17126	-0.08404	2.7501	-2.75093	27.593
843.03	6.16417	6.12436	-6.15211	-6.151	-0.06689	2.74002	-2.73834	27.583
853.03	6.14209	6.12793	-6.15599	-6.15494	-0.06521	2.74684	-2.71209	27.5833
863.03	6.12628	6.11966	-6.15571	-6.14809	-0.07073	2.72995	-2.73435	27.57254
873.03	6.14577	6.12525	-6.1546	-6.12213	-0.06431	2.75401	-2.72931	27.56336
883.03	6.12763	6.12124	-6.15756	-6.15442	-0.0682	2.73678	-2.7265	27.56936
893.03	6.12887	6.11585	-6.14228	-6.15723	-0.07024	2.73308	-2.7311	27.5596
903.03	6.12873	6.12112	-6.14185	-6.15928	-0.06569	2.73329	-2.70831	27.5566
913.03	6.13648	6.12588	-6.14044	-6.0978	-0.0648	2.7365	-2.72725	27.5496
923.03	6.1261	6.12222	-6.13913	-6.11539	-0.06934	2.72195	-2.73393	27.5436
933.03	6.14133	6.13231	-6.14981	-6.15817	-0.06288	2.74019	-2.71156	27.5336
943.03	6.13315	6.12337	-6.15715	-6.15059	-0.06622	2.73393	-2.73066	27.5336
953.03	6.13514	6.11447	-6.16486	-6.14647	-0.07792	2.73381	-2.75113	27.5336
963.03	6.14882	6.13538	-6.15941	-6.15201	-0.06489	2.73538	-2.72951	27.5336

Table E Displacement of characteristic points in the Y-direction of expansive soil samples and changes in moisture content of soil samples with drying time.

t	D1	D2	D3	D4	D5	D6	D7	W
min	mm	mm	mm	mm	mm	mm	mm	%
0	0	0	0	0	0	0	0	35.58648
10.05	0.01514	0.01853	-0.10486	0.22853	-0.00912	-0.00242	-0.00853	35.38767

20.08333	0.00811	0.02292	-0.11189	0.23292	-0.04189	0.00403	-0.02849	35.18887
30.11667	-0.01207	0.0777	-0.13207	0.2877	-0.10305	-0.03206	-0.10048	34.99006
40.16667	-0.03091	0.127	-0.15091	0.337	-0.16696	-0.06204	-0.15646	34.79125
50.2	-0.04873	0.187	-0.16873	0.397	-0.18936	-0.0722	-0.18339	34.59245
60.25	-0.06016	0.227	-0.18016	0.437	-0.23832	-0.1067	-0.25003	34.49304
70.28333	-0.11436	0.277	-0.23436	0.487	-0.28434	-0.11422	-0.27562	34.29423
80.33333	-0.16258	0.337	-0.28258	0.547	-0.31697	-0.15158	-0.31461	34.09543
90.36667	-0.21466	0.397	-0.33466	0.607	-0.34125	-0.14237	-0.38678	33.89662
100.4167	-0.26586	0.437	-0.38586	0.647	-0.40121	-0.16956	-0.41844	33.79722
110.4667	-0.32149	0.4975	-0.44149	0.7075	-0.44009	-0.2183	-0.42792	33.59841
120.5	-0.4155	0.5628	-0.5355	0.7728	-0.45005	-0.24543	-0.42417	33.3996
130.55	-0.50265	0.61258	-0.62265	0.82258	-0.49406	-0.30611	-0.4485	33.3002
140.5833	-0.58134	0.67289	-0.70134	0.88289	-0.52065	-0.3607	-0.46255	33.10139
150.6333	-0.67133	0.71258	-0.79133	0.92258	-0.55208	-0.39193	-0.49045	32.90258
160.6667	-0.7469	0.77159	-0.8669	0.98159	-0.56418	-0.42424	-0.49586	32.80318
170.7167	-0.84269	0.82156	-0.96269	1.03156	-0.61021	-0.47883	-0.5362	32.60437
180.75	-0.93258	0.86258	-1.05258	1.07258	-0.62236	-0.50827	-0.54036	32.40557
190.8	-1.01457	0.89258	-1.13457	1.10258	-0.63017	-0.53903	-0.54288	32.30616
200.8333	-1.11595	0.93258	-1.23595	1.14258	-0.67626	-0.59325	-0.59669	32.10736
210.8833	-1.19258	0.98256	-1.30258	1.19256	-0.66204	-0.59834	-0.57106	32.00795
220.9167	-1.2586	1.04236	-1.3786	1.25236	-0.63906	-0.59217	-0.55467	31.80915
230.9667	-1.31258	1.09258	-1.43258	1.30258	-0.653	-0.63536	-0.5635	31.70974

241	-1.36582	1.1538	-1.48582	1.3638	-0.65975	-0.6469	-0.55894	31.51093
251.05	-1.4258	1.20258	-1.5458	1.41258	-0.64665	-0.63429	-0.54464	31.41153
261.0833	-1.47596	1.25892	-1.59596	1.46892	-0.66085	-0.64831	-0.56326	31.21272
271.1167	-1.51239	1.29358	-1.63239	1.50358	-0.66886	-0.66013	-0.56129	31.01392
281.1667	-1.55268	1.33258	-1.67268	1.54258	-0.68914	-0.671	-0.59238	30.91451
291.2	-1.61237	1.39528	-1.73237	1.60528	-0.68516	-0.67627	-0.58547	30.71571
301.25	-1.65823	1.42368	-1.77823	1.63368	-0.70501	-0.67869	-0.61123	30.6163
311.2833	-1.68269	1.44237	-1.80269	1.65237	-0.71794	-0.67564	-0.6271	30.4175
321.3333	-1.72596	1.47662	-1.84596	1.68662	-0.72544	-0.65603	-0.64244	30.31809
331.3667	-1.75238	1.49325	-1.87238	1.70325	-0.75754	-0.64101	-0.6766	30.11928
341.4167	-1.80236	1.55236	-1.92236	1.76236	-0.75852	-0.62715	-0.68904	30.01988
351.45	-1.83258	1.60228	-1.95258	1.81228	-0.76754	-0.60685	-0.68892	29.92048
361.5	-1.8524	1.634	-1.9724	1.844	-0.79411	-0.61471	-0.71949	29.82107
371.5333	-1.89024	1.65075	-2.01024	1.86075	-0.79966	-0.62262	-0.72306	29.72167
381.5833	-1.88046	1.66069	-2.00046	1.87069	-0.81922	-0.62724	-0.73605	29.52286
391.6167	-1.87948	1.67187	-1.99948	1.88187	-0.82425	-0.62757	-0.74381	29.42346
401.6667	-1.86531	1.68468	-1.98531	1.89468	-0.83371	-0.62506	-0.74566	29.32406
411.7	-1.8818	1.65924	-2.0018	1.86924	-0.85886	-0.64795	-0.76636	29.22465
421.7333	-1.87614	1.67768	-1.99614	1.88768	-0.84742	-0.64141	-0.76005	29.12525
431.7833	-1.87405	1.68058	-1.99405	1.89058	-0.8545	-0.64426	-0.76048	29.02584
441.8167	-1.85403	1.68248	-1.97403	1.89248	-0.85307	-0.64177	-0.76219	29.02584
451.85	-1.87648	1.68022	-1.99648	1.89022	-0.86639	-0.65384	-0.77432	28.92644

461.9	-1.86449	1.68915	-1.98449	1.89915	-0.8626	-0.64347	-0.7702	28.82704
471.9333	-1.87675	1.67796	-1.99675	1.88796	-0.87415	-0.65859	-0.77993	28.72763
481.9667	-1.88859	1.69643	-2.00859	1.90643	-0.88107	-0.64841	-0.78225	28.68763
492.0167	-1.87224	1.6889	-1.99224	1.8989	-0.87602	-0.64955	-0.77754	28.62823
502.05	-1.87153	1.69694	-1.99153	1.90694	-0.87493	-0.65002	-0.77386	28.58823
512.1	-1.87781	1.67192	-1.99781	1.88192	-0.87768	-0.65364	-0.78952	28.52883
522.1333	-1.86806	1.68294	-1.98806	1.89294	-0.88971	-0.65575	-0.7938	28.48883
532.1667	-1.87171	1.67987	-1.99171	1.88987	-0.87762	-0.64892	-0.79254	28.42942
542.2167	-1.88773	1.68341	-2.00773	1.89341	-0.89062	-0.67382	-0.77855	28.38942
552.25	-1.87233	1.69252	-1.99233	1.90252	-0.89836	-0.65673	-0.7806	28.33002
562.3	-1.86515	1.68895	-1.98515	1.89895	-0.87527	-0.65743	-0.7821	28.30002
572.3333	-1.87809	1.68241	-1.99809	1.89241	-0.89228	-0.6622	-0.80513	28.26002
582.3667	-1.86508	1.6845	-1.98508	1.8945	-0.89519	-0.66108	-0.82183	28.23062
592.4167	-1.88052	1.6914	-2.00052	1.9014	-0.88724	-0.66268	-0.79428	28.19062
602.45	-1.88888	1.67345	-2.00888	1.88345	-0.90449	-0.67336	-0.80886	28.16062
612.5	-1.89815	1.66948	-2.01815	1.87948	-0.89819	-0.67344	-0.80283	28.13121
622.5333	-1.8829	1.68115	-2.0029	1.89115	-0.89656	-0.66649	-0.80089	28.10121
632.5833	-1.89932	1.67564	-2.01932	1.88564	-0.90605	-0.68392	-0.82161	28.07121
642.6167	-1.89749	1.6647	-2.01749	1.8747	-0.90422	-0.68092	-0.80905	28.06121
652.65	-1.88337	1.67704	-2.00337	1.88704	-0.90025	-0.67249	-0.80827	28.03181
662.7	-1.88025	1.67734	-2.00025	1.88734	-0.89671	-0.66604	-0.8125	28.00181
672.7333	-1.88744	1.69414	-2.00744	1.90414	-0.87573	-0.66313	-0.80208	27.97818

682.7833	-1.87388	1.68177	-1.99388	1.89177	-0.89794	-0.66553	-0.80568	27.95181
692.8167	-1.89675	1.66133	-2.01675	1.87133	-0.89576	-0.68542	-0.81493	27.91181
692.8667	-1.86914	1.68665	-1.98914	1.89665	-0.89886	-0.66043	-0.79316	27.88241
702.9	-1.89426	1.68095	-2.01426	1.89095	-0.91499	-0.67923	-0.81881	27.85241
712.9333	-1.88021	1.68307	-2.00021	1.89307	-0.89607	-0.66503	-0.80356	27.83241
722.9833	-1.88338	1.68293	-2.00338	1.89293	-0.89974	-0.67306	-0.8089	27.817
733.0167	-1.87616	1.67756	-1.99616	1.88756	-0.90124	-0.66585	-0.80661	27.79241
743.0333	-1.87289	1.68145	-1.99289	1.89145	-0.89602	-0.6594	-0.79865	27.76241
753.03	-1.8866	1.68141	-2.0066	1.89141	-0.90139	-0.68591	-0.81945	27.72241
763.03	-1.88682	1.6858	-2.00682	1.8958	-0.8903	-0.66855	-0.79644	27.693
773.03	-1.87591	1.69159	-1.99591	1.90159	-0.88919	-0.66467	-0.80354	27.673
783.03	-1.88071	1.68916	-2.00071	1.89916	-0.8946	-0.65583	-0.79395	27.653
793.03	-1.86857	1.67268	-1.98857	1.88268	-0.90158	-0.66078	-0.81838	27.643
803.03	-1.88589	1.69294	-2.00589	1.90294	-0.89829	-0.67739	-0.81229	27.6423
813.03	-1.88388	1.68077	-2.00388	1.89077	-0.90039	-0.66613	-0.80936	27.633
823.03	-1.88784	1.67699	-2.00784	1.88699	-0.9058	-0.67126	-0.81423	27.613
833.03	-1.888	1.68048	-2.008	1.89048	-0.92941	-0.68717	-0.8248	27.593
843.03	-1.88318	1.6757	-2.00318	1.8857	-0.89546	-0.66741	-0.80638	27.583
853.03	-1.8867	1.68451	-2.0067	1.89451	-0.90347	-0.67288	-0.80332	27.5833
863.03	-1.87703	1.69078	-1.99703	1.90078	-0.89178	-0.66399	-0.79705	27.57254
873.03	-1.86712	1.67277	-1.98712	1.88277	-0.90575	-0.64672	-0.8167	27.56336
883.03	-1.8809	1.68104	-2.0009	1.89104	-0.90505	-0.67079	-0.80833	27.56936

893.03	-1.88145	1.68429	-2.00145	1.89429	-0.90226	-0.66879	-0.80637	27.5596
903.03	-1.88094	1.68619	-2.00094	1.89619	-0.89356	-0.66707	-0.80748	27.5566
913.03	-1.87957	1.68169	-1.99957	1.89169	-0.89979	-0.66954	-0.80465	27.5496
923.03	-1.87971	1.68791	-1.99971	1.89791	-0.88887	-0.66755	-0.804	27.5436
933.03	-1.8752	1.68857	-1.9952	1.89857	-0.89491	-0.66413	-0.79992	27.5336
943.03	-1.8968	1.67082	-2.0168	1.88082	-0.91204	-0.68408	-0.81993	27.5336
953.03	-1.88929	1.6796	-2.00929	1.8896	-0.90823	-0.67709	-0.81683	27.5336
963.03	-1.90084	1.67499	-2.02084	1.88499	-0.91145	-0.68252	-0.81763	27.5336

Table F Displacement of characteristic points in the Z-direction of expansive soil samples and changes in moisture content of soil samples with drying time.

t<br>min	D1<br>mm	D2<br>mm	D3<br>mm	D4<br>mm	D5<br>mm	D6<br>mm	D7<br>mm	W<br>%
0	0	0	0	0	0	0	0	35.58648
10.05	0.05	0.07	0.015	0.0775	0.04	0.02	0.122	35.38767
20.08333	0.13	0.145	0.0359	0.0984	0.07952	0.04	0.19862	35.18887
30.11667	0.22	0.225	0.05698	0.11948	0.11236	0.05	0.25	34.99006
40.16667	0.29	0.315	0.06738	0.12988	0.15986	0.13	0.33	34.79125
50.2	0.375	0.385	0.14738	0.20988	0.20288	0.22	0.42	34.59245
60.25	0.465	0.47	0.23738	0.25698	0.23698	0.29	0.49	34.49304
70.28333	0.575	0.56	0.29585	0.35835	0.2757	0.375	0.575	34.29423
80.33333	0.705	0.65	0.36238	0.42488	0.31568	0.465	0.665	34.09543
90.36667	0.825	0.8	0.48238	0.54488	0.3424	0.575	0.775	33.89662
100.4167	0.915	0.92	0.59238	0.65488	0.37893	0.705	0.905	33.79722

110.4667	1.025	1.01	0.72238	0.81568	0.43262	0.825	1.025	33.59841
120.5	1.105	1.12	0.84238	0.90488	0.51262	0.915	1.115	33.3996
130.55	1.215	1.2	0.93238	0.99488	0.62262	1.025	1.225	33.3002
140.5833	1.295	1.3268	1.04238	1.10488	0.70262	1.105	1.305	33.10139
150.6333	1.355	1.39	1.12238	1.18488	0.76262	1.215	1.415	32.90258
160.6667	1.425	1.45	1.23238	1.29488	0.83262	1.26358	1.46358	32.80318
170.7167	1.475	1.52	1.31238	1.37488	0.88262	1.28238	1.49896	32.60437
180.75	1.455	1.57	1.39238	1.45488	0.86262	1.15488	1.4568	32.40557
190.8	1.415	1.55	1.44238	1.50488	0.82262	1.075	1.435	32.30616
200.8333	1.345	1.51	1.49238	1.54893	0.75262	0.995	1.345	32.10736
210.8833	1.265	1.44	1.47238	1.53488	0.67262	0.928	1.265	32.00795
220.9167	1.165	1.36	1.43238	1.49488	0.57262	0.846	1.165	31.80915
230.9667	1.075	1.26	1.36238	1.42488	0.48262	0.75626	1.075	31.70974
241	0.995	1.17	1.28238	1.34488	0.40262	0.68126	0.995	31.51093
251.05	0.928	1.09	1.18238	1.24488	0.33562	0.59624	0.928	31.41153
261.0833	0.846	1.023	1.09238	1.15488	0.25362	0.50624	0.846	31.21272
271.1167	0.75626	0.941	1.01238	1.07488	0.16388	0.42659	0.75626	31.01392
281.1667	0.68126	0.85126	0.94538	1.00788	0.06737	0.31626	0.68126	30.91451
291.2	0.59624	0.77626	0.86338	0.92588	-0.01766	0.28097	0.59624	30.71571
301.25	0.50624	0.69124	0.77364	0.83614	-0.10765	0.19097	0.50624	30.6163
311.2833	0.42659	0.60124	0.69864	0.76114	-0.1873	0.11132	0.42659	30.4175
321.3333	0.31626	0.52159	0.61362	0.67612	-0.29763	9.93E-04	0.31626	30.31809
331.3667	0.22658	0.41126	0.52362	0.58612	-0.38731	-0.08868	0.22658	30.11928

341.4167	0.09624	0.32158	0.44397	0.50647	-0.51765	-0.21903	-0.09544	30.01988
351.45	-0.00572	0.29893	0.33364	0.39614	-0.63372	-0.33509	-0.2115	29.92048
361.5	-0.12572	0.25655	0.24396	0.30646	-0.77372	-0.47509	-0.3515	29.82107
371.5333	-0.21572	0.24262	0.11362	0.17612	-0.84372	-0.54509	-0.4215	29.72167
381.5833	-0.32872	0.23399	0.18637	0.11637	-0.95672	-0.65809	-0.5345	29.52286
391.6167	-0.45872	0.20455	0.13287	0.06287	-1.08672	-0.78809	-0.66451	29.42346
401.6667	-0.59272	0.18146	-0.03113	-0.10113	-1.22072	-0.92209	-0.7985	29.32406
411.7	-0.73172	0.08246	-0.23013	-0.30013	-1.41972	-1.12109	-0.9975	29.22465
421.7333	-0.85772	0.01646	-0.29613	-0.36613	-1.48572	-1.18709	-1.0635	29.12525
431.7833	-0.98259	-0.10841	-0.421	-0.491	-1.61059	-1.31197	-1.18838	29.02584
441.8167	-1.11872	-0.24454	-0.55713	-0.62713	-1.7357	-1.43707	-1.31348	29.02584
451.85	-1.23972	-0.36554	-0.67813	-0.74813	-1.86772	-1.56909	-1.4455	28.92644
461.9	-1.32972	-0.45554	-0.76813	-0.83813	-1.95772	-1.65909	-1.5355	28.82704
471.9333	-1.44872	-0.57454	-0.88713	-0.95713	-2.07672	-1.7781	-1.65451	28.72763
481.9667	-1.56892	-0.69474	-1.00733	-1.07733	-2.19692	-1.8983	-1.77471	28.68763
492.0167	-1.69489	-0.82071	-1.1333	-1.2033	-2.32289	-2.02426	-1.90067	28.62823
502.05	-1.81784	-0.94366	-1.25625	-1.32625	-2.44584	-2.14722	-2.02363	28.58823
512.1	-1.93626	-1.06634	-1.37893	-1.44893	-2.54368	-2.24506	-2.12147	28.52883
522.1333	-2.04272	-1.16854	-1.48113	-1.55113	-2.67072	-2.37209	-2.2485	28.48883
532.1667	-2.15472	-1.48605	-1.79864	-1.68964	-2.76983	-2.4712	-2.34761	28.42942
542.2167	-2.26572	-1.56334	-1.87593	-1.75893	-2.86598	-2.56736	-2.44377	28.38942
552.25	-2.39179	-1.57761	-1.8302	-1.9002	-3.01979	-2.72116	-2.59757	28.33002

562.3	-2.51272	-1.63854	-1.95113	-2.02113	-3.14072	-2.84209	-2.7185	28.30002
572.3333	-2.62872	-1.7743	-2.08689	-2.15689	-3.25672	-2.95809	-2.8345	28.26002
582.3667	-2.76018	-2.05634	-2.36893	-2.25993	-3.38818	-3.08955	-2.96597	28.23062
592.4167	-2.88672	-2.17804	-2.49063	-2.38163	-3.51472	-3.21609	-3.0925	28.19062
602.45	-3.00572	-2.22421	-2.4368	-2.5068	-3.62639	-3.32777	-3.20418	28.16062
612.5	-3.13572	-2.27154	-2.58413	-2.65413	-3.77372	-3.47509	-3.3515	28.13121
622.5333	-3.26472	-2.37999	-2.69258	-2.76258	-3.86289	-3.56427	-3.44068	28.10121
632.5833	-3.38375	-2.46669	-2.77928	-2.84928	-4.01175	-3.71312	-3.58953	28.07121
642.6167	-3.49372	-2.58568	-2.89827	-2.96827	-4.08786	-3.78924	-3.66565	28.06121
652.65	-3.58372	-2.70568	-3.01827	-3.08827	-4.20786	-3.90924	-3.78565	28.03181
662.7	-3.66372	-2.78954	-3.10213	-3.17213	-4.29172	-3.99309	-3.8695	28.00181
672.7333	-3.71372	-2.83954	-3.15213	-3.22213	-4.34172	-4.04309	-3.9195	27.97818
682.7833	-3.72258	-2.8484	-3.16099	-3.23099	-4.35058	-4.05497	-3.93138	27.95181
692.8167	-3.71569	-2.84151	-3.1541	-3.2241	-4.34369	-4.04808	-3.92449	27.91181
692.8667	-3.72157	-2.84739	-3.15998	-3.22998	-4.34957	-4.05396	-3.93037	27.88241
702.9	-3.71568	-2.8415	-3.15409	-3.22409	-4.34368	-4.04807	-3.92448	27.85241
712.9333	-3.72129	-2.84711	-3.1597	-3.2297	-4.34929	-4.05368	-3.93009	27.83241
722.9833	-3.7157	-2.84152	-3.15411	-3.22411	-4.3437	-4.04809	-3.9245	27.817
733.0167	-3.70987	-2.83569	-3.14828	-3.21828	-4.33787	-4.04226	-3.91867	27.79241
743.0333	-3.71985	-2.84567	-3.15826	-3.22826	-4.34785	-4.05224	-3.92865	27.76241
753.03	-3.71237	-2.83819	-3.15078	-3.22078	-4.34037	-4.04476	-3.92117	27.72241
763.03	-3.72028	-2.8461	-3.15869	-3.22869	-4.34828	-4.05267	-3.92908	27.693

773.03	-3.7099	-2.83572	-3.14831	-3.21831	-4.3379	-4.04228	-3.91869	27.673
783.03	-3.71124	-2.83706	-3.14965	-3.21965	-4.33924	-4.04362	-3.92004	27.653
793.03	-3.71024	-2.83606	-3.14865	-3.21865	-4.33824	-4.04262	-3.91904	27.643
803.03	-3.71236	-2.83818	-3.15077	-3.22077	-4.34036	-4.04475	-3.92116	27.6423
813.03	-3.70988	-2.8357	-3.14829	-3.21829	-4.33788	-4.04226	-3.91867	27.633
823.03	-3.71236	-2.83818	-3.15077	-3.22077	-4.34036	-4.04475	-3.92116	27.613
833.03	-3.7259	-2.82818	-3.14077	-3.21077	-4.33036	-4.04043	-3.91684	27.593
843.03	-3.70257	-2.82839	-3.14098	-3.21098	-4.33057	-4.04064	-3.91705	27.583
853.03	-3.7159	-2.82172	-3.13431	-3.20431	-4.3239	-4.03397	-3.91038	27.5833
863.03	-3.71236	-2.83818	-3.15077	-3.22077	-4.34036	-4.05043	-3.92684	27.57254
873.03	-3.72003	-2.84585	-3.15844	-3.22844	-4.34803	-4.0581	-3.93451	27.56336
883.03	-3.71	-2.83582	-3.14841	-3.21841	-4.338	-4.04807	-3.92448	27.56936
893.03	-3.71236	-2.83818	-3.15077	-3.22077	-4.34036	-4.05043	-3.92684	27.5596
903.03	-3.71236	-2.83818	-3.15077	-3.22077	-4.34036	-4.05043	-3.92684	27.5566
913.03	-3.70985	-2.83567	-3.14826	-3.21826	-4.33785	-4.04793	-3.92434	27.5496
923.03	-3.71024	-2.83606	-3.14865	-3.21865	-4.33824	-4.04831	-3.92472	27.5436
933.03	-3.72	-2.84582	-3.15841	-3.22841	-4.348	-4.05808	-3.93449	27.5336
943.03	-3.71985	-2.84567	-3.15826	-3.22826	-4.34785	-4.05793	-3.93434	27.5336
953.03	-3.72157	-2.84739	-3.15998	-3.22998	-4.34957	-4.05964	-3.93605	27.5336
963.03	-3.71568	-2.8415	-3.15409	-3.22409	-4.34368	-4.05375	-3.93017	27.5336

Table G Secretary of isotropic shrinkage of red clay soil samples.

Soil sample size	lateral shrinkage	Longitudinal shrinkage	Vertical shrinkage	Percentage reduction of area	Volume shrinkage
mm	%	%	%	%	%
200*10*10	5.6	8.4	9.8	13.53	22
200*30*10	8.7	9.9	11.7	17.63	27.2
200*50*10	11.1	11.52	13.6	20.83	32.2
200*30*30	10.3	11.44	15.5	20.47	32.7
200*30*50	10.5	11.85	17.1	20.84	34.2

TableH Secretary of isotropic shrinkage on expansive soil samples.

Soil sample size	lateral shrinkage	Longitudinal shrinkage	Vertical shrinkage	Percentage reduction of area	Volume shrinkage
mm	%	%	%	%	%
200*10*10	3.81	4.42	6.69	9.23	14
200*30*10	5.74	6.12	8.62	11.51	17.77
200*50*10	7.83	8.2	10.72	15.39	24.46
200*30*30	6.79	8.14	12.46	14.38	25.05
200*30*50	6.95	8.96	14.37	15.29	27.46

Table I Comparison of lateral shrinkage between red clay and expansive soil.

Soil sample size	The lateral shrinkage of red clay	The lateral shrinkage of expansive soil
mm	%	%
20*10*10	5.6	3.81
20*30*10	8.7	5.74
20*50*10	11.1	7.83
20*30*30	10.2	6.79
20*30*50	10.5	6.95

Table J Comparison of longitudinal shrinkage between red clay and expansive soil.

Soil sample size mm	The longitudinal shrinkage ratio of red clay %	The longitudinal shrinkage ratio of expansive soil %
20*10*10	8.4	4.42
20*30*10	9.9	6.12
20*50*10	11.52	8.2
20*30*30	11.44	8.14
20*30*50	11.85	8.96

Table K Comparison of vertical shrinkage between red clay and expansive soil.

Soil sample size mm	The vertical shrinkage of red clay %	The vertical shrinkage of expansive soil %
20*10*10	9.8	6.69
20*30*10	11.7	8.62
20*50*10	13.6	10.72
20*30*30	15.5	12.46
20*30*50	17.1	14.37

Table L porosity of red clay soil samples varies with moisture content

moisture content %	Void ratio
51.23	1.01637
50.81	1.01217
50.387	1.00794
49.967	1.00374
49.067	0.99474
48.167	0.98574
47.277	0.97684
46.387	0.96794
45.497	0.95904
44.607	0.95014
43.697	0.94104
42.777	0.93184
41.857	0.92264

40.937	0.91344
40.017	0.90424
39.087	0.89494
38.167	0.88574
37.237	0.87644
36.307	0.86714
35.377	0.85784
34.447	0.84854
33.517	0.83924
32.587	0.82994
31.657	0.82064
30.727	0.81134
29.797	0.80204
28.867	0.79274
27.937	0.78344
27.007	0.77414
26.077	0.76684
25.147	0.76227
24.217	0.75729
23.287	0.75311
22.357	0.74952
21.637	0.74651
20.937	0.74391
20.247	0.74186
19.587	0.7398
18.947	0.73818
18.327	0.73685
17.717	0.73583
17.117	0.73412
16.537	0.73412
15.977	0.73424
15.427	0.73399
14.897	0.73399
14.377	0.73401
13.877	0.73446
13.397	0.73457
12.937	0.73416
12.487	0.73357
12.057	0.7337
11.657	0.73411
11.277	0.73457
10.907	0.73399
10.557	0.7337
10.237	0.73416

9.937	0.73412
9.657	0.7341
9.407	0.73401
9.197	0.73414
9.017	0.7341
8.877	0.73399
8.777	0.73457
8.677	0.73413
8.577	0.7341
8.477	0.73102
8.427	0.7341
8.377	0.73412
8.327	0.73467
8.287	0.734
8.247	0.734
8.207	0.734
8.177	0.73412
8.157	0.73457
8.147	0.73416

Table M Porosity from expansive soils varies with moisture content.

moisture content %	Void ratio
35.58648	0.86439
35.38767	0.8624
35.18887	0.86041
34.99006	0.85843
34.79125	0.85644
34.59245	0.85445
34.49304	0.85346
34.29423	0.85147
34.09543	0.84948
33.89662	0.84749
33.79722	0.8465
33.59841	0.84451
33.3996	0.84252
33.3002	0.84153
33.10139	0.83954
32.90258	0.83755
32.80318	0.83656
32.60437	0.83457
32.40557	0.83258
32.30616	0.83159

32.10736	0.8296
32.00795	0.8286
31.80915	0.82662
31.70974	0.82562
31.51093	0.82363
31.41153	0.82266
31.21272	0.82107
31.01392	0.81999
30.91451	0.81867
30.71571	0.81698
30.6163	0.81538
30.4175	0.8133
30.31809	0.81271
30.11928	0.81096
30.01988	0.80976
29.92048	0.809
29.82107	0.80737
29.72167	0.80684
29.52286	0.80466
29.42346	0.80357
29.32406	0.80209
29.22465	0.80064
29.12525	0.79951
29.02584	0.7992
29.02584	0.799
28.92644	0.79837
28.82704	0.79766
28.72763	0.79704
28.68763	0.7964
28.62823	0.79605
28.58823	0.7956
28.52883	0.79538
28.48883	0.795
28.42942	0.7944
28.38942	0.79401
28.33002	0.7938
28.30002	0.79361
28.26002	0.79303
28.23062	0.79257
28.19062	0.79211
28.16062	0.79208
28.13121	0.79205
28.10121	0.79201
28.07121	0.79183

28.06121	0.79163
28.03181	0.79116
28.00181	0.79084
27.97818	0.79062
27.95181	0.79062
27.91181	0.79062
27.88241	0.79062
27.85241	0.79062
27.83241	0.79062
27.817	0.79062
27.79241	0.79062
27.76241	0.79062
27.72241	0.79062
27.693	0.79062
27.673	0.79062
27.653	0.79062
27.643	0.79062
27.6423	0.79062
27.633	0.79062
27.613	0.79062
27.593	0.79062
27.583	0.79062
27.5833	0.79062
27.57254	0.79062
27.56336	0.79062
27.56936	0.79062
27.5596	0.79062
27.5566	0.79062
27.5496	0.79062
27.5436	0.79062
27.5336	0.79062
27.5336	0.79062
